# Formation and Accumulation of Acetaldehyde and Strecker Aldehydes during Red Wine Oxidation

**DOI:** 10.3389/fchem.2018.00020

**Published:** 2018-02-14

**Authors:** Mónica Bueno, Almudena Marrufo-Curtido, Vanesa Carrascón, Purificación Fernández-Zurbano, Ana Escudero, Vicente Ferreira

**Affiliations:** ^1^Instituto de Ciencias de la Vid y del Vino, Universidad de La Rioja-CSIC-Gobierno de La Rioja, Logroño, Spain; ^2^Laboratory for Flavor Analysis and Enology, Department of Analytical Chemistry, Faculty of Sciences, Instituto Agroalimentario de Aragón, IA2, Universidad de Zaragoza-CITA, Universidad de Zaragoza, Zaragoza, Spain

**Keywords:** amino acids, sulfur dioxide, quinones, α-dicarbonyls, iron, off-odors, oxidative deterioration, PLS models

## Abstract

The main aim of the present work is to study the accumulation of acetaldehyde and Strecker aldehydes (isobutyraldehyde, 2-methylbutanal, isovaleraldehyde, methional, phenylacetaldehyde) during the oxidation of red wines, and to relate the patterns of accumulation to the wine chemical composition. For that, eight different wines, extensively chemically characterized, were subjected at 25°C to three different controlled O_2_ exposure conditions: low (10 mg L^−1^) and medium or high (the stoichiometrically required amount to oxidize all wine total SO_2_ plus 18 or 32 mg L^−1^, respectively). Levels of volatile aldehydes and carbonyls were then determined and processed by different statistical techniques. Results showed that young wines (<2 years-old bottled wines) hardly accumulate any acetaldehyde regardless of the O_2_ consumed. In contrast, aged wines (>3 years-old bottled wines) accumulated acetaldehyde while their content in SO_2_ was not null, and the aged wine containing lowest polyphenols accumulated it throughout the whole process. Models suggest that the ability of a wine to accumulate acetaldehyde is positively related to its content in combined SO_2_, in epigallocatechin and to the mean degree of polymerization, and negatively to its content in Aldehyde Reactive Polyphenols (ARPs) which, attending to our models, are anthocyanins and small tannins. The accumulation of Strecker aldehydes is directly proportional to the wine content in the amino acid precursor, being the proportionality factor much higher for aged wines, except for phenylacetaldehyde, for which the opposite pattern was observed. Models suggest that non-aromatic Strecker aldehydes share with acetaldehyde a strong affinity toward ARPs and that the specific pattern of phenylacetaldehyde is likely due to a much reduced reactivity toward ARPs, to the possibility that diacetyl induces Strecker degradation of phenyl alanine and to the potential higher reactivity of this amino acid to some quinones derived from catechin. All this makes that this aldehyde accumulates with intensity, particularly in young wines, shortly after wine SO_2_ is depleted.

## Introduction

Oxygen is a key factor to achieve wine optimum quality (Ugliano, 2013). Some of the improvements linked to an optimized use of oxygen are color stabilization (Ribéreau-Gayon et al., 1983; Atanasova et al., 2002a; Cano-Lopez et al., 2008; Wirth et al., 2010), the balance of astringency, bitterness, and mouthfeel (Cejudo-Bastante et al., 2011; Chira et al., 2012), and the decrease of vegetal and green aromas (Ortega Heras et al., 2008; Cejudo-Bastante et al., 2011). However, an excessive exposure to oxygen can lead to the development of yellow and brown colors (Singleton and Kramling, 1976) and to wine aroma deterioration (Ugliano, 2013; Ferreira et al., 2014). This is related to the development of oxidation-related aldehydes (Cullere et al., 2007) with notes of rancid, honey, raisins, dried fruit, or cooked potato (Escudero et al., 2000a,b; Ferreira et al., 2003).

**Figure 1:**
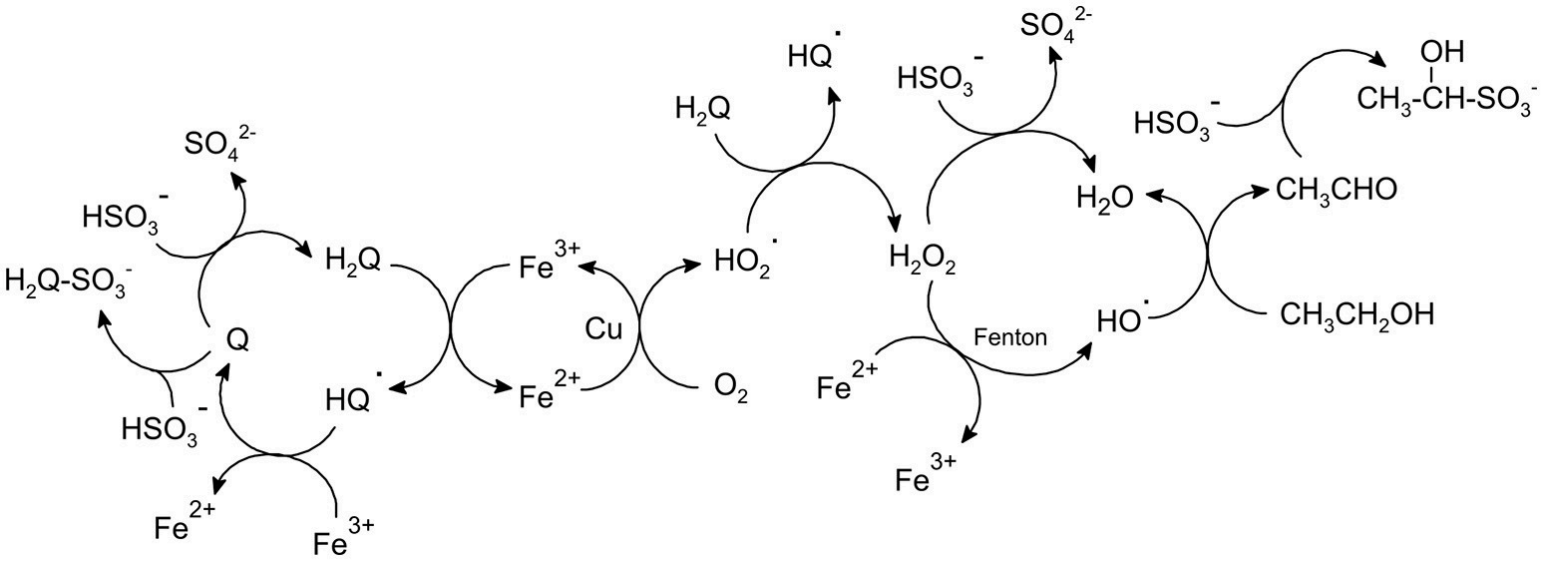
Oxidation mechanism and SO_2_ reactions proposed in red wine. Adapted from Danilewicz (2003, 2011, 2014); Danilewicz and Wallbridge (2010), and Waterhouse et al. (2016). Q, quinone; HQ, semiquinone; H_2_Q, *o*-diphenol.

The reactions that take place in wine when it is exposed to oxygen have been deeply studied (Singleton, 1987; Atanasova et al., 2002a; Waterhouse and Laurie, 2006; Danilewicz, 2007; Danilewicz et al., 2008; Dimkou et al., 2011; Oliveira et al., 2011b; Laurie et al., 2012; Ugliano, 2013). The accepted mechanism (Figure 1) supports that once oxygen is dissolved in wine, its activation is carried out by metal ions, particularly iron and copper in lower oxidation states (Fe^2+^ and Cu^+^), supposedly forming the yet undetected hydroperoxy radical (${\text{HO}}_{2}^{•}$) (Danilewicz and Wallbridge, 2010; Danilewicz, 2011; Waterhouse et al., 2016) and Fe^3+^. This oxidized ion reacts with the catechol group of some of the many wine phenolic compounds, forming back Fe^2+^ (Danilewicz, 2014). The radical ${\text{HO}}_{2}^{∙}$ subsequently reacts with the phenols to form hydrogen peroxide (H_2_O_2_) which can produce Fenton reaction by reacting with Fe^2+^ (Danilewicz, 2003; Waterhouse et al., 2016) to give the powerful oxidant HO^∙^ -hydroxyl radical-, which is able to oxidize nearly all types of organic compounds (Waterhouse et al., 2016). Sulfur dioxide plays a key pivotal role in this scheme, taking part in two of the main reactions. It removes H_2_O_2_ by reducing it to water (Danilewicz, 2003) and hence blocking Fenton reaction, and it also can react with the quinones either reducing them back to catechols or forming a sulfonate as nucleophilic addition product (Waterhouse et al., 2016).

**Figure 2:**
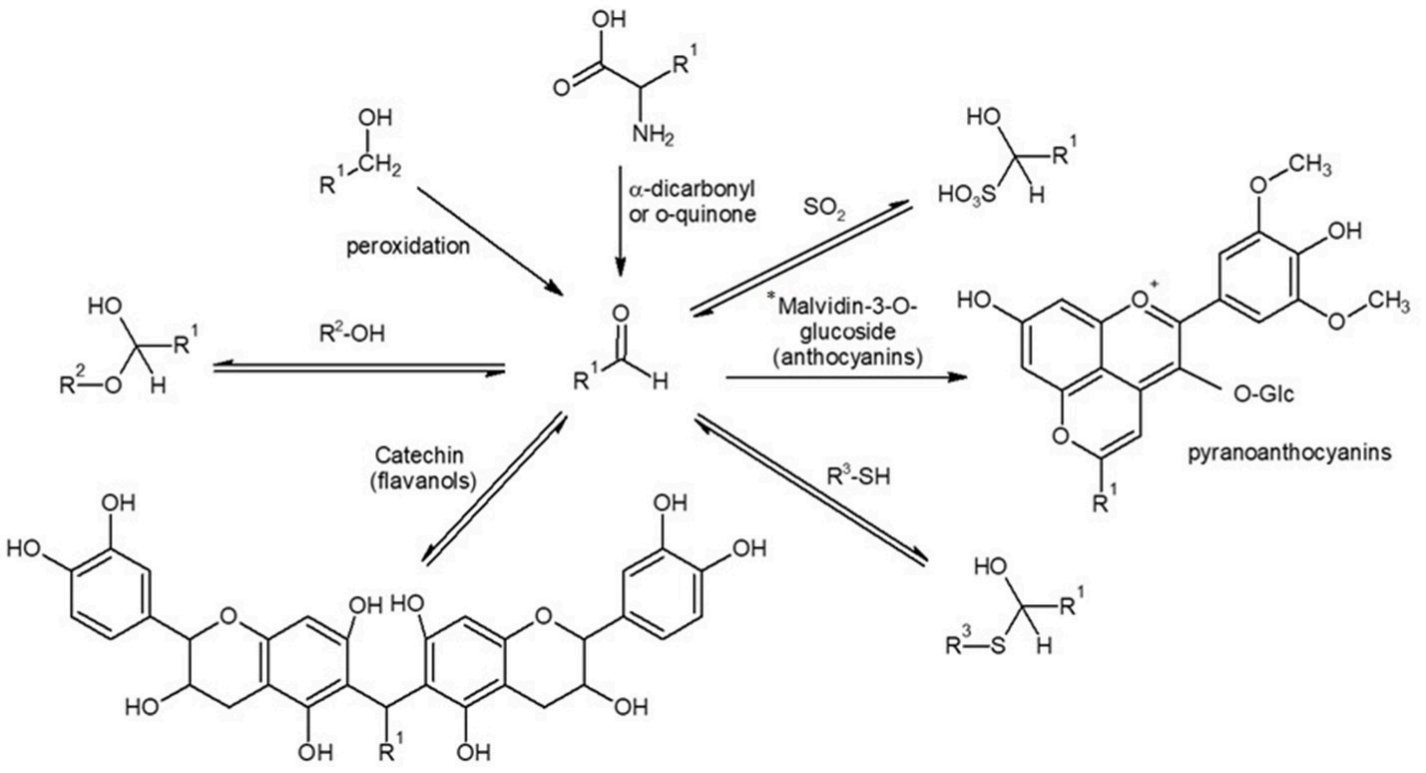
Formation and reaction of aldehydes in wine. Adapted from Grant-Preece et al. (2013) and Waterhouse et al. (2016). *Only aldehydes that can exist in a stable -enol form can produce the pyranoanthocyanins.

The major oxidation-related aldehyde is acetaldehyde, which is the major oxidation by-product of the Fenton oxidation of wine, as seen in Figure 1. From the sensory point of view, however, methional and phenylacetaldehyde play a major role (Cullere et al., 2007). These aldehydes, together with the other Strecker aldehydes (isobutyraldehyde, 2-methylbutanal, isovaleraldehyde, methional, phenylacetaldehyde) will be similarly formed from the corresponding precursor alcohols by peroxidation, as suggested by different authors (Wildenradt and Singleton, 1974; Escudero et al., 2000b; San Juan et al., 2012), but they can be also formed via Strecker degradation of the corresponding precursor amino acid (Rizzi, 2006; Grant-Preece et al., 2013). In such reaction, the amino acid reacts with an α-dicarbonyl, such as diacetyl (Pripis-Nicolau et al., 2000) or an *o*-quinone (Singleton, 1987) which will be particularly abundant during oxidation (Monforte et al., 2017) (Figure 2).

Nevertheless, understanding the formation of aldehydes becomes further complicated by the numbers of reversible and irreversible chemical processes in which these compounds are involved. Aldehydes can reversibly bind to SO_2_ forming α-hydroxyalkylsulfonates (Figures 1, 2) (de Azevedo et al., 2007; Grant-Preece et al., 2013; Bueno et al., 2014). The α-hydroxyalkylsulfonates of acetaldehyde (*K*_*a*_ = 485 × 10^3^; de Azevedo et al., 2007) and Strecker aldehydes (isobutyraldehyde *K*_*a*_ = 2.8 × 10^3^, isovaleraldehyde *K*_*a*_ = 29 × 10^3^, 2-methylbutanal *K*_*a*_ = 2.6 × 10^3^, methional *K*_*a*_ = 50 × 10^3^, and phenylacetaldehyde *K*_*a*_ = 17 × 10^3^; Bueno et al., 2014) can be present in non-oxidized wines, acting as an odorless reservoir of oxidation-related aldehydes which will be released during wine oxidation, as SO_2_ is depleted and equilibria shifts (Bueno et al., 2016).

The electrophile character of aldehydes makes them reactive to other wine nucleophiles (Figure 2). They can reversibly bind also to thiols such as glutathione or cysteine to give α-hydroxysulfides (Lienhard and Jencks, 1966; Sonni et al., 2011; Baert et al., 2015a), which seem to play an important role in beer flavor instability (Baert et al., 2015b). Similar reversible reactions between aldehydes and the amino group of the amino acids to form imines have been studied in synthetic medium, and do not seem to be relevant (Baert et al., 2015a). Aldehydes can also react with alcohols resulting in acetals (Schneider et al., 1998; Ferreira et al., 2002; Camara et al., 2003).

Moreover, some of the most important reactions of aldehydes in wine are those with phenolic compounds (Figure 2). The A ring of flavonoids is a phloroglucinol moiety with different positions with nucleophilic strength that can react with carbonyls, especially with those that could have a stable -enol form, such as acetaldehyde, forming a wide range of products, such as pyranoanthocyanins (Bakker and Timberlake, 1997; Vivar-Quintana et al., 1999; de Freitas and Mateus, 2011; Marquez et al., 2013) that are compounds formed from anthocyanidins with a new pyrano ring. For example vitisin B is the cycloaddition product of acetaldehyde and malvidin-3-*O*-glucoside. In this case the reaction takes place through the enol tautomer of acetaldehyde which attacks at the C-4 and C-5 positions of the anthocyanin and further dehydrates and oxidizes (de Freitas and Mateus, 2011). Another family of reaction products between aldehydes and flavanols or among aldehydes, flavanols and anthocyanins are dimers or longer polymers in which aldehydes act as bridges; for example in the case of acetaldehyde, there is a 8,8-methylmethine bridge, commonly named ethyl bridge (Fulcrand et al., 1996, 1997; Es-Safi et al., 1999, 2000, 2002; Escribano-Bailon et al., 2001; Atanasova et al., 2002b; Duenas et al., 2006). In this last case, the reaction is thought to take place via direct nucleophilic attack on the 8 position to introduce a 1-hydroxylethyl group, which further dehydrates and suffers the nucleophilic attack from a 8 (or 6) position of the second molecule (Nave et al., 2010).

This broad reactivity of aldehydes takes place concurrently with their formation when levels of free SO_2_ are low, which implies that the study of the formation of aldehydes requires to ensure oxidation conditions in which wine SO_2_ is completely depleted. These conditions were not reached in previous studies, in which the observed increases in free aldehydes were mostly caused by release from hydroxyalkylsulfonates (Bueno et al., 2016). In this paper the main goals are to study the accumulation of acetaldehyde and Strecker aldehydes during the oxidation of wine and to assess the influence of the different compositional factors on such accumulation. For that, eight different red wines were extensively characterized and subsequently subjected to three different controlled O_2_ exposure conditions during which the levels of total aldehydes were measured.

## Materials and methods

### Wines

Eight commercial bottled Spanish red wines of different vintages (between 2009 and 2014) and made from four different grape varieties, Garnacha (Grenache), Tempranillo Merlot, and Cabernet-Sauvignon, were used in the study. Details of the samples together with some compositional parameters are shown in Table 1.

**Table 1:** Wines analyzed in the experiment including origin, varietal composition, age, and some basic chemical data.

**Wine code**	**Region**	**Grape variety^a^**	**Vintage**	**Oak aging (months)**	**Bottle aging (months) Approx**.	**Ethanol % (v/v)**	**Total SO_2_ (mg L^−1^)**	**Free SO_2_ (mg L^−1^)**	**pH**	**Abs420**	**Abs520**	**Abs620**	**CI^b^**	**TPI^c^**	**Folin-Ciocalteu (mg L^−1^ GAE)^d^**
**AGED WINES**															
SL	Rioja	T. M. G	2011	12	3.00	13.5	75.6	24.7	3.51	3.82	4.24	0.95	9.01	61.8	2435.1
TS	Toro	T	2012	14	1.84	14.5	76.8	33.7	3.60	3.79	4.27	0.99	9.05	63.1	2591.4
BL	Rioja	T	2010	20	3.34	13.5	40.0	13.3	3.61	4.11	4.40	1.15	9.65	58.2	2304.7
CH	Campo de Borja	G	2009	15	4.75	14.0	31.2	2.61	3.32	5.04	5.35	1.25	11.6	71.5	2654.8
**YOUNG WINES**															
MF	Rioja	T	2014	0	1.00	13.5	25.6	10.6	3.58	3.31	4.37	1.14	8.82	48.8	2067.2
TP	Campo de Borja	G	2013	5	1.59	15.0	28.0	9.83	3.26	4.09	5.73	1.33	11.1	50.8	2051.1
HV	Calatayud	G	2014	0	1.00	14.5	12.8	13.6	3.29	3.15	4.06	0.91	8.12	53.0	2384.4
BS	Campo de Borja	G. T. CS	2014	0	1.00	13.5	32.0	11.3	3.31	3.08	4.29	0.91	8.28	46.9	2094.1

a Grape Varierty, T, Tempranillo; M, Merlot; G, Garnacha; CS, Cabernet-Sauvignon;

b Color Intensity, expressed as (A420 + A520 + A620).

c Total Polyphenol Index, expressed in absorbance × 100.

d *GAE, Gallic Acid Equivalents*.

### Wine oxidation procedure

**Figure 3:**
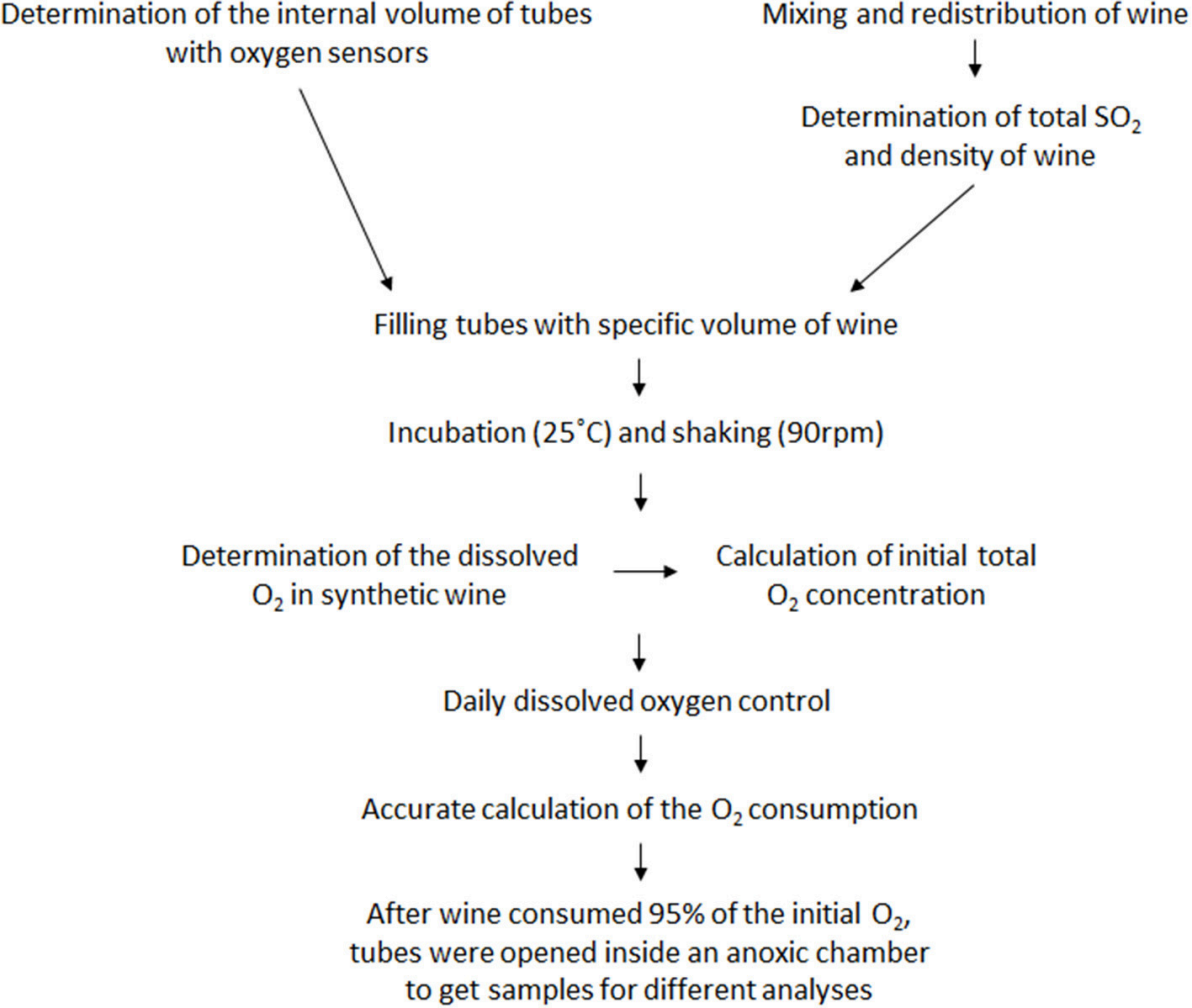
Wine oxidation experiment following the procedure described by Marrufo-Curtido et al. (2018).

Wines were subjected to three different controlled oxygen exposure conditions (R1, R2, and R3) in duplicate following the procedure described in Marrufo-Curtido et al. (2018) (Figure 3). In such procedure, perfectly controlled volumes of sterile filtered (OIV, 2015) wine are enclosed with perfectly controlled volumes of air in air-tight tubes (60 mL nominal internal volume from WIT-France, Bordeaux) containing PSt3 oxygen sensors (Nomacorc S.A., Thimister-Clermont, Belgium). The accurate internal volume and weight of each one of the tubes were previously determined by standard calibration practices.

In order to avoid bottle effects, five bottles of each one of the eight wines used in the experiment were opened, mixed in a large beaker, filtered and redistributed back in the five bottles. The bottles were purged for 1 min with a 415 mL min^−1^ flow of argon, were further resealed with a Nomacorc Select 300 CP closure and were stored in the fridge until the beginning of the experiment. One bottle was then used to measure wine density and total sulfur dioxide, parameters required in the procedure.

The experiment begins by opening a bottle, transferring a volume of wine to a graduated cylinder and carefully closing the bottle with a wine saver vacuvin closure (IIC Brands, The Netherlands). Then, a specific volume of wine was quickly transferred to the calibrated WIT tube. The tube was immediately weighted and closed. The weight of the filled tube is used to determine the exact volume of wine contained in the tube (V_l_). The exact volume of headspace (V_g_) was estimated by subtracting V_l_ from V_int_, the truly internal volume of the tube. The time elapsed between uncorking and closing the WIT tube with the wine was less than 2 min. The tubes were then left in a thermostatic bath Grant OLS23 with orbital shaking (90 rpm) at 25 ± 0.1°C to ensure that liquid and gas phases were always in equilibrium. Oxygen was measured with an oxygen analyzer Fibox 3 LCD trace from Nomacorc SA. Oxygen level was monitored each 30 min during the first 3 h, and then once per day. Each experiment was considered complete when the wine consumed 95% of the initial oxygen. At that time, the WIT tubes were opened inside a glove box without oxygen (oxygen < 0.002%) to get samples for analysis avoiding the wine to be further oxidized.

The total amount of oxygen contained initially in the tube was estimated from calibrated tubes filled with volumes of synthetic wine following exactly the same procedures used for the wines. In these cases, the dissolved oxygen in the liquid phase was measured after half an hour inside the incubator shaker to ensure a perfect equilibration. Those concentrations in the liquid phase of equilibrated synthetic wines were R1: 5.0 ± 0.13, R2: 6.35 ± 0.04, and R3: 6.75 ± 0.02 mg L^−1^ (means of four replicates each). Then, knowing that the saturation concentration of O_2_ in our wine models completely equilibrated with air (21% v/v in Oxygen) was 7.73 ± 0.07 mg L^−1^ (*n* = 4), the exact volume of headspace (V_g_) and of liquid (V_l_) within the tubes, and assuming that oxygen in gas and liquid phases are in equilibrium, it is possible to estimate the initial total amount of oxygen. In R1 experiments, the total amount of oxygen contained in the tube corresponded to 9–11 mg of oxygen per liter of wine; in R2 experiments, the total amount within the tubes ranged from 22 to 35 mg of oxygen per liter of wine. In this case, the amount delivered to each wine was 18 mg L^−1^ plus the stoichiometrically required amount to oxidize all its total SO_2_. Similarly, in R3 experiments, the oxygen given to each wine was 32 mg L^−1^ plus the stoichiometrically required amount to oxidize all its total SO_2_ so that total amounts of oxygen within the tubes ranged from 35 to 53 mg of oxygen per liter of wine.

### Solvents and chemicals

Sodium metabisulfite 99% (Na_2_S_2_O_5_), tartaric acid (99%), glycerol (99.5%), 1,2-propanediol (99.5%), sodium hydroxide (98%), ortho phosphoric acid (85%), hydrogen peroxide 3% stabilized w/v VINIKIT, indicator 4,4, mixed (methyl red-methylene blue) VINIKIT, sodium hydroxide 0.01 mol L^−1^ VINIKIT were from Panreac (Barcelona, Spain). Dichloromethane, ethanol and methanol for gas chromatography analyses were purchased from Merk (Darmstadt, Germany). Methanol and acetonitrile of HPLC quality were obtained from Fluka Analytical (Buchs, Switzerland). Hydrochloric acid 37%, formic acid and ammonium formate high purity grade were purchased from VWR Prolabo (Fontenay sous Bois, France). Phloroglucinol (≥99%), ascorbic acid (≥99%), acetaldehyde (≥99.5%), 2-chloroethanol (≥ 99.0%), methyl 2-methylbutyrate (≥ 99%), 2-butanol (≥ 99%), glyoxal 40% in water, Folin-Ciocalteu's phenol reagent, sodium carbonate (≥ 99%), gallic acid (≥ 99%), and (+)-catechin (≥ 99%) were supplied by Sigma-Aldrich (Madrid, Spain). Standards and reagents for aroma compounds and amino acids determination were purchased from Sigma-Aldrich, Fluka, Panreac, Lancaster (Eastgate, UK), PolyScience (Niles, IL, USA), ChemService (West Chester, PA, USA), and Firmenich (Switzerland), and details of the chemicals have been already reported (Singleton et al., 1998; Ortega et al., 2001; Hernández-Orte et al., 2003; Ribéreau-Gayon et al., 2006; OIV, 2009b; Herrero et al., 2011; Bueno et al., 2014; Grindlay et al., 2014; Vallverdu-Queralt et al., 2016; Carrascón et al., 2017). Water was purified in a Milli-Q system from Millipore (Bedford, Germany).

### Analytical characterization

Initial wines (R0) were analyzed in duplicate for total acetaldehyde and total odor-active carbonyls, as well as for free and total SO_2_, pH, color parameters, total polyphenol index and Folin-Ciocalteu index, phenolic, and tannin composition, major aroma compounds, metals, and amino acids.

Final samples after oxidation procedures were analyzed for total acetaldehyde, total odor-active carbonyls, as well as for free (only for the low exposure) and total SO_2_, color parameters, total polyphenol, and major aroma compounds.

#### Sulfur dioxide determination

Free sulfur dioxide was determined by headspace gas chromatography with a mass spectrometer detector (HS-GC-MS) in a QP 2010 GC-MS from Shimadzu (Kyoto, Japan) following the procedure described in a previous work (Carrascón et al., 2017). For the analysis, 4.5 mL of sample acidified with 500 μL of ortho-phosphoric acid (85%) were incubated at 40 ± 0.1°C for 15 min. Then, 400 μL of the headspace were injected in a split/splitless injector. External calibration curve in model wine containing known amounts of sulfur dioxide, obtained by dissolving sodium metabisulfite (Na_2_S_2_O_5_) was prepared to quantify this compound.

For total sulfur dioxide determination, the aspiration-oxidation method recommended by the OIV (International Organization of Vine and Wine) was used (OIV, 2009b). Attending to such procedure, 10 mL of sample acidified with 5 mL of 25% H_3_PO_4_ and heated to 100°C were bubbled with air for 15 min (with a flow of 600 ± 12 mL min^−1^). The SO_2_ released was collected in pear shaped flask containing 3 mL of neutralized hydrogen peroxide (3%) with two drops of mixed indicator (methyl red—methylene blue) in which sulfur dioxide was completely oxidized to sulfuric acid, turning the color of the solution from green to purple. The sulfuric acid formed was titrated with standardized 0.01 M NaOH.

#### Total acetaldehyde determination

Total acetaldehyde was determined by gas chromatography with flame ionization detection (GC-FID) by injection of 1 μL of wine sample spiked with 2-butanol (100 mg L^−1^) as internal standard (Supplementary Figure 1). The method is based on breaking the adducts directly in the injector port. A GC 8000 series from Fisons Instrument (Ipswich, United Kingdom) with a DB-WAX (30 m × 0.53 mm of i.d. x 2 μm) capillary column from J&W Scientific (Agilent Technologies, Santa Clara, CA) were used. The injector was kept at 250°C and the split ratio was 1:4. Hydrogen was used as carried gas and the pressure was kept at 27.5 kPa. The temperature program was 50°C for 5 min and then raised to 220°C in 10 min. The FID temperature was 250°C.

The linearity of the method was obtained by the analysis of synthetic wines (5 g L^−1^ tartaric acid, 12% ethanol, 1.5% propane-1,2-diol, 10 g L^−1^ glycerin, pH 3.5) containing known amounts of acetaldehyde and plotting the corresponding peak areas (normalized by that of the internal standard methyl 2-butanol) vs. the mass of acetaldehyde. The linear dynamic range spanned from the method detection limit to more than 120 mg L^−1^ with determination coefficient better than 0.998. Detection limit, defined as three times the *SD* of the noise of the base line close to the peak was estimated to be 0.22 mg L^−1^. Method repeatability was determined by replicated analysis of a real wine containing 21.2 mg L^−1^ acetaldehyde and was estimated to be 4.1% (*n* = 8). Reproducibility was estimated by the replicate analysis of that wine in different days and was 5.3% (*n* = 4). The ability of the method to break acetaldehyde-SO_2_ adducts was experimentally verified by comparing the areas obtained in the analysis of synthetic wines with 100 mg L^−1^ acetaldehyde containing different amounts of SO_2_ (from 0 to 200 mg L^−1^) and previously incubated 12 h. No significant differences were found. The overall accuracy of the method was studied in a standard recovery experiment in which two different red wines were spiked with 20 mg L^−1^ of acetaldehyde, left to stand 12 h, and analyzed in triplicate. Determined amounts of acetaldehyde were 19.0 ± 1.6 and 19.6 ± 1.3 mg L^−1^ not significantly different from the added value.

#### Determination of total odor-active carbonyls

The determination by headspace-SPME-GC-MS of total (free plus bound) forms of different odor-active carbonyls such as isobutyraldehyde, 2-methylbutanal, isovaleraldehyde, methional, phenylacetaldehyde, and diacetyl in wine is described in the method proposed by Bueno et al. (2014). The wines were opened inside an oxygen free chamber from Jacomex (Dagneux, France), 10 mL were transferred into a 20 mL headspace vial, and were then spiked with methyl 2-methylbutyrate (187 μg L^−1^) as internal standard and with 6 g L^−1^ of glyoxal for breaking complexes. Vials were then closed, taken out of the glove box and incubated in a laboratory oven at 50 ± 0.5°C for 6 h in order to break carbonyl-bisulfite complexes. Strict anoxia conditions are essential to prevent any oxidation. Then, carbonyls in the headspace were preconcentrated on a PDMS/DVB fiber (Supleco-Spain, Madrid, Spain) and were further analyzed on a GC-MS equipped with a quadrupole in SIM mode (Supplementary Figure 2).

#### Spectrophotometric measurements

For color determination, absorbances at wavelengths 420, 520, and 620 nm of undiluted wine samples were measured using glass cells with optical paths of 1, 2, or 5 mm, taking the measurement which provided absorbance readings between 0.3 and 0.7, as recommended by the OIV (2009a). Total Phenolic Index (TPI) was determined as OD 280 as described by Ribéreau-Gayon et al. (2006). Folin-Ciocalteau assay was performed following the method described by Singleton et al. (1998) using 1 cm quartz cuvettes. All the absorbance measurements were taken using an UV–vis spectrophotometer UV-17000 Pharma Spec from Shimadzu (Kyoto, Japan).

#### Metal analyses

A direct five-fold aqueous dilution of wine was analyzed by inductively coupled plasma-mass spectrometry with collision/reaction cell (CCT-ICP-MS) as described by Grindlay et al. (2014) using rhodium as internal standard. Metals quantified were iron, copper, zinc, and manganese.

#### Amino acid analyses

Strecker amino acids (valine, isoleucine, leucine, phenylalanine, and methionine) plus cysteine were determined by HPLC with fluorescence detector according to the method reported by Hernández-Orte et al. (2003). The method involves derivatization with aminoquinolyl-N-hydrosysuccinimidyl carbamate (AQC). A quaternary HPLC system Waters 2695 from Waters (Milford, MA) with a fluorescence detector ProStar 363 from Varian (Walnut Creek, CA) were used.

#### Determination of major aroma compounds

Major aroma compounds such as isobutanol, isoamyl alcohol, methionol, β-phenylethanol, and acetic acid were determined using a variation of the method published by Ortega et al. (2001). The strategy followed a liquid-liquid microextraction with dichloromethane and uses several internal standards to correct for matrix effects (recoveries above 95% in all cases). 2-Butanol was used as internal standard for isobutanol, 4-methyl-2-pentanol for isoamyl alcohol, and benzyl alcohol and 4-hydroxy-4-methyl-2-pentanone for methionol, and β-phenylethanol, all of them spiked at 1.5 mg L^−1^ to the wine. Analyses were carried out using a GC-3800 from Varian (Walnut Creek, CA) equipped with a flame ionization detector (FID). The column used was a DB-WAX from J&W (Folsom, CA) 30 m × 0.32 mm × 0.5 mm film thickness, preceded by a silica precolumn from Agilent Technologies (Santa Clara, CA), 3 m × 0.32 mm i.d. The carrier gas was He at 2.2 mL min^−1^. Two microliters were injected in split mode (1:20). Injector and detector were both kept at 250°C. The temperature program: 40°C for 5 min, then raised at 4°C min^−1^ up to 102°C, 2°C min^−1^ up to 112°C, 3°C min^−1^ up to 125°C, this temperature was kept for 5 min, 3°C min^−1^ up to 160°C, 6°C min^−1^ up to 200°C, and this temperature was kept for 30 min.

#### Analyses of phenolics

Phenolic acids, flavanols, and anthocyanins were determined by ultra-high performance liquid chromatography (UPLC) using mass spectrometry (MS) and a diode array detector (DAD). Wines were analyzed in triplicate in positive and negative mode following the procedure described by Vallverdu-Queralt et al. (2016) in a Waters Acquity UPLC–DAD system (Waters, Milford, MA, USA) with a reverse phase Acquity BEH C18 column (150 mm length, 1 mm internal diameter, 1.7 μm particle size) from Waters (Milford, MA, USA). The spectrometer hyphenated to the UPLC–DAD system was a Bruker Daltonics Amazon (Bruker, Darmstadt, Germany) mass spectrometer.

Composition of condensed tannins was studied by phloroglucinolysis reaction (acid-catalyzed depolymerization in the presence of a nucleophilic agent) following the modifications of the procedure publish by Kennedy and Jones (2001) as described by Carrascón et al. (2018). The reaction was performed in triplicate and the depolymerized samples were analyzed by UPLC-DAD-MS at 280 nm. The molar ratio of total amount of depolymerized subunits to terminal subunits provides the mean degree of polymerization (mDP) (Ducasse et al., 2010). Quantification was done in equivalents of catechin, epicatechin, epigallocatechin, and epicatechin-3-O-gallate at 280 nm.

### Data treatment and statistical analysis

Levels of carbonyls obtained in the different samples were analyzed by repeated measures ANOVA using SPSS v15. (SPSS Inc., IBM Company, Chicago, IL, USA) for assessing the effects of oxygen level and wine sample. Correlation studies were directly carried out with Excel 2013 (Microsoft, Washington, USA). The uncertainty of each mean was calculated taking into account experimental uncertainty, measured via experimental replicates, and the analytical uncertainty, measured via analytical replicates.

Principal Component Analysis was performed using XLSTAT (Addinsoft, version 2015). Partial Least Square Regression analysis was carried out using The Unscrambler 9.7 (CAMO Software AS, Oslo, Norway). PLS modeling was carried out using cross-validation criteria. In this strategy, the model is built in iterations leaving out one of the samples in each iteration. The predicted result for the sample left out is us to compute the model error. The process is repeated until every sample has been left out once; then all prediction residuals are combined to compute the validation residual variance and the RMSEP (Root mean square prediction error).

## Results and discussion

Four samples of each wine were obtained in the experiment carried out in the present work: the initial control (samples R0) and three oxidized specimens (samples R1, R2, and R3). R1 samples were exposed to a level of O_2_ roughly equivalent to two air-saturations, while R2 and R3 were exposed to levels of O_2_, respectively equivalent to 18 or 32 mg L^−1^ plus the stoichiometrically required amount to oxidize all the total SO_2_ initially present in each particular wine.

Levels of carbonyls found in the different samples are summarized in Table 2. In all cases data correspond to total carbonyls, since methods used for their determination involved a previous step to break reversible adducts, such as those formed with sulfur dioxide. Levels of carbonyls can be further related to the total amount of O_2_ given to the wine or, more interestingly, to the amount of O_2_ not invested in the oxidation of wine total SO_2_ (*O*_2_ *not SO*_2_). This is calculated by subtracting from the total amount of O_2_ taken by a sample, the amount of O_2_ which would have been taken by the SO_2_ consumed by that specific wine assuming a 2:1 molar ratio.

**Table 2:** Levels of total Strecker aldehydes (μg L^−1^), acetaldehyde, and diacetyl (mg L^−1^) measured in the eight wines in the oxidation experiment.

**Wine and O_2_ dose**	***O_2_ not SO_2_*^#^ (mg L^−1^)**	Total O_2_^*^ (mg L^−1^)	**Isobutyraldehyde**	**Isovaleraldehyde**	**2-methylbutanal**	**Methional**	**Phenylacetaldehyde**	**Acetaldehyde**	**Diacetyl**
SL R0	0 ± 0	0 ± 0	37.8 ± 1.0^c^	50.9 ± 1.9^b^	12.5 ± 0.2^c^	29.2 ± 0.4^d^	59.4 ± 13.3^b^	19.6 ± 0.5^d^	4.20 ± 0.12^a^
SL R1	2.9 ± 0.3	10.1 ± 0.3	38.9 ± 1.0^c^	56.9 ± 0.7^b^	12.6 ± 0.1^c^	35.5 ± 1.2^c^	67.9 ± 1.7^b^	25.1 ± 0.7^c^	4.29 ± 0.21^a^
SL R2	14.8 ± 0.5	33.7 ± 0.5	48.1 ± 2.5^b^	68.9 ± 2.3^a^	20.1 ± 0.2^b^	48.4 ± 0.9^b^	80.7 ± 1.0^a^	31.8 ± 0.5^a^	3.90 ± 0.25^a^
SL R3	29.8 ± 0.7	48.7 ± 0.7	57.4 ± 0.6^a^	67.0 ± 1.1^a^	22.5 ± 0.1^a^	62.2 ± 0.8^a^	90.3 ± 2.2^a^	28.4 ± 0.9^b^	3.75 ± 0.27^a^
TS R0	0 ± 0	0 ± 0	25.3 ± 0.6^c^	33.0 ± 2.0^b^	12.3 ± 1.5^b^	23.4 ± 0.0^b^	34.5 ± 7.7^b^	17.8 ± 0.7^c^	5.58 ± 0.16^a^
TS R1	2.8 ± 0.6	11.3 ± 0.6	24.9 ± 0.6^c^	33.7 ± 0.6^b^	11.4 ± 0.0^b^	22.8 ± 1.6^b^	28.8 ± 1.9^b^	23.7 ± 1.2^b^	5.03 ± 0.02^ab^
TS R2	16.2 ± 0.6	35.4 ± 0.6	31.9 ± 0.0^b^	40.7 ± 0.1^a^	16.0 ± 0.6^a^	35.1 ± 0.6^a^	48.7 ± 0.2^a^	26.8 ± 0.2^a^	4.52 ± 0.20^ab^
TS R3	33.8 ± 1.4	53.0 ± 1.4	37.4 ± 0.4^a^	42.1 ± 0.8^a^	17.9 ± 0.4^a^	36.1 ± 1.1^a^	52.8 ± 1.1^a^	26.2 ± 0.1^a^	3.73 ± 0.17^b^
BL R0	0 ± 0	0 ± 0	29.9 ± 0.7^c^	50.0 ± 1.6^d^	10.2 ± 1.1^c^	19.2 ± 0.5^d^	34.3 ± 7.7^c^	13.9 ± 0.1^d^	2.34 ± 0.10^a^
BL R1	5.3 ± 0.2	10.6 ± 0.2	33.4 ± 0.7^c^	57.7 ± 4.1^c^	12.1 ± 0.3^c^	27.9 ± 2.8^c^	46.2 ± 4.8^b^	21.3 ± 0.6^c^	2.43 ± 0.30^a^
BL R2	18.9 ± 0.3	28.9 ± 0.3	40.9 ± 0.9^b^	74.8 ± 4.7^b^	18.0 ± 0.9^b^	43.8 ± 4.0^b^	74.7 ± 1.6^a^	23.6 ± 1.6^b^	2.30 ± 0.15^a^
BL R3	32.4 ± 0.0	42.4 ± 0.0	51.5 ± 2.1^a^	82.6 ± 0.2^a^	23.4 ± 1.5^a^	55.4 ± 3.8^a^	86.7 ± 5.7^a^	31.6 ± 0.6^a^	2.29 ± 0.20^a^
CH R0	0 ± 0	0 ± 0	50.0 ± 7.5^c^	37.4 ± 6.7^d^	19.7 ± 1.6^c^	32.2 ± 2.2^d^	62.3 ± 23.3^c^	15.9 ± 0.1^c^	0.43 ± 0.01^a^
CH R1	7.0 ± 0.2	10.5 ± 0.2	50.2 ± 1.8^c^	45.8 ± 4.3^c^	20.9 ± 1.4^c^	41.8 ± 1.2^c^	85.1 ± 4.0^b^	24.3 ± 0.9^b^	0.41 ± 0.01^a^
CH R2	18.9 ± 0.1	26.7 ± 0.1	71.7 ± 0.9^b^	90.5 ± 0.3^b^	36.9 ± 0.0^b^	82.6 ± 2.4^b^	145.6 ± 1.4^a^	27.6 ± 0.7^a^	0.50 ± 0.01^a^
CH R3	34.2 ± 1.0	42.0 ± 1.0	91.0 ± 3.5^a^	112.3 ± 4.4^a^	45.3 ± 2.1^a^	102.1 ± 1.0^a^	154.8 ± 6.6^a^	26.7 ± 0.2^a^	0.62 ± 0.03^a^
MF R0	0 ± 0	0 ± 0	17.4 ± 0.4^c^	30.7 ± 0.2^ab^	7.1 ± 0.2^c^	22.1 ± 0.0^b^	32.7 ± 1.2^c^	11.3 ± 0.7^a^	7.12 ± 0.24^a^
MF R1	6.5 ± 0.1	10.1 ± 0.1	15.9 ± 0.4^c^	29.6 ± 0.2^b^	6.5 ± 0.1^c^	18.6 ± 0.8^b^	38.0 ± 1.0^c^	14.3 ± 0.9^b^	6.14 ± 0.23^a^
MF R2	17.2 ± 0.0	23.6 ± 0.0	24.3 ± 0.8^b^	30.6 ± 0.8^b^	9.8 ± 0.4^b^	29.9 ± 0.4^a^	112.1 ± 2.4^b^	17.5 ± 0.6^a^	3.69 ± 0.56^b^
MF R3	28.6 ± 0.2	35.0 ± 0.2	31.8 ± 0.1^a^	36.7 ± 0.0^a^	13.0 ± 0.1^a^	33.0 ± 1.0^a^	136.1 ± 14.0^a^	17.1 ± 0.1^a^	2.94 ± 0.05^b^
TP R0	0 ± 0	0 ± 0	24.8 ± 0.4^bc^	51.2 ± 0.2^a^	7.9 ± 0.3^b^	24.6 ± 0.0^b^	90.6 ± 3.8^b^	13.1 ± 0.4^a^	14.12 ± 0.34a
TP R1	4.8 ± 0.3	9.4 ± 0.3	21.3 ± 0.7^c^	42.8 ± 0.3^b^	7.3 ± 0.5^b^	20.3 ± 1.6^b^	72.9 ± 4.9^c^	13.5 ± 0.1^b^	13.82 ± 2.65a
TP R2	17.2 ± 0.1	24.2 ± 0.1	28.6 ± 0.4^ab^	42.2 ± 0.4^b^	10.0 ± 0.0^a^	32.8 ± 3.3^a^	161.4 ± 8.4^a^	13.5 ± 0.1^b^	7.44 ± 0.89b
TP R3	30.8 ± 0.9	37.8 ± 0.9	32.4 ± 0.5^a^	42.0 ± 1.4^b^	11.3 ± 0.4^a^	35.2 ± 2.3^a^	154.0 ± 34.7^a^	13.3 ± 0.4^b^	5.88 ± 0.14b
HV R0	0 ± 0	0 ± 0	23.4 ± 1.0^b^	27.8 ± 2.1^ab^	6.3 ± 0.2^bc^	25.9 ± 0.0^bc^	41.3 ± 1.8^c^	17.9 ± 0.9^a^	4.60 ± 0.21^a^
HV R1	9.7 ± 0.3	9.8 ± 0.3	17.8 ± 1.2^c^	21.4 ± 0.5^b^	5.0 ± 0.0^c^	20.2 ± 1.1^c^	37.5 ± 5.0^c^	18.6 ± 0.4^ab^	3.55 ± 0.01^ab^
HV R2	19.3 ± 0.2	22.5 ± 0.2	25.2 ± 1.5^b^	23.8 ± 1.8^b^	7.2 ± 0.3^b^	28.3 ± 0.2^ab^	109.2 ± 6.4^b^	15.7 ± 0.0^c^	1.93 ± 0.22^b^
HV R3	32.2 ± 0.3	35.4 ± 0.3	31.2 ± 1.4^a^	30.9 ± 0.8^a^	9.8 ± 0.2^a^	33.7 ± 0.4^a^	135.6 ± 12.1^a^	17.0 ± 0.0^bc^	1.82 ± 0.03^b^
BS R0	0 ± 0	0 ± 0	17.4 ± 0.3^bc^	32.6 ± 0.5^a^	5.2 ± 0.2^bc^	21.2 ± 0.0^bc^	33.1 ± 0.7^b^	17.0 ± 1.0^a^	9.89 ± 0.22^a^
BS R1	8.8 ± 0.2	10.8 ± 0.2	16.6 ± 0.4^c^	32.3 ± 1.1^a^	4.9 ± 0.0^c^	17.5 ± 0.4^c^	36.3 ± 1.9^b^	16.7 ± 0.6^a^	9.48 ± 0.27^a^
BS R2	18.8 ± 0.3	26.8 ± 0.3	21.1 ± 1.0^b^	28.9 ± 0.3^a^	6.7 ± 0.1^ab^	25.3 ± 1.7^ab^	101.6 ± 0.5^a^	14.8 ± 0.3^a^	4.65 ± 0.69^b^
BS R3	32.0 ± 1.3	40.0 ± 1.3	28.4 ± 0.5^a^	33.0 ± 0.8^a^	9.2 ± 0.1^a^	31.8 ± 2.1^a^	107.9 ± 5.4^a^	16.4 ± 0.1^a^	3.86 ± 0.17^b^

# O_2_ consumed not invested in the oxidation of wine SO_2_.

* Total oxygen consumed.

a, b, c, d Different letters in a column indicate significant differences (p ≤ 0.05) between dose of oxygen for the same wine.

*R0, initial wine; R1, low; R2, medium; R3, high oxygen exposure*.

### Fate of acetaldehyde

**Figure 4:**
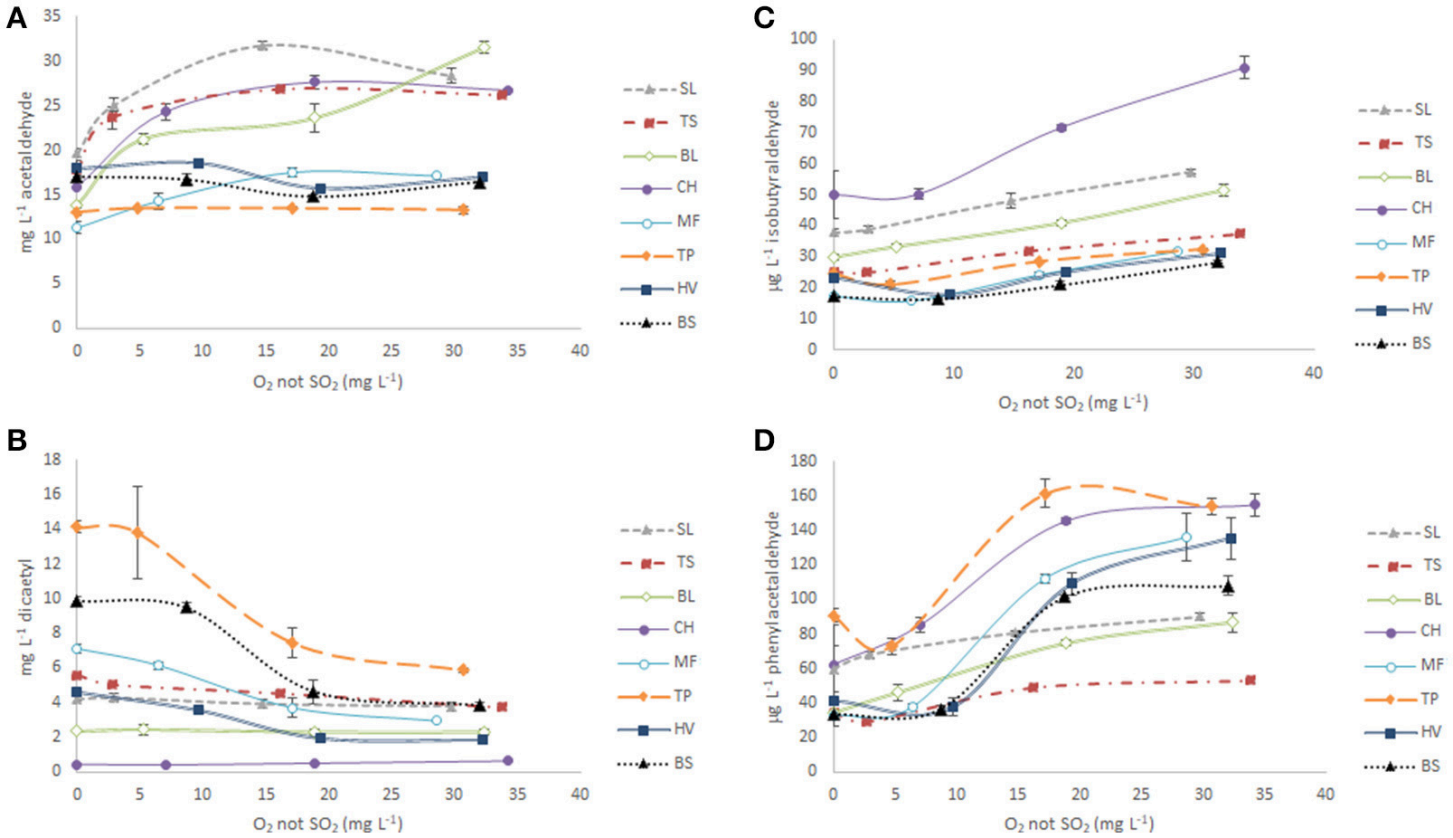
Evolution of the amounts of total aldehydes present in each wine exposed to different levels of oxygen. The X axis represents the amount of O_2_ consumed not invested in the oxidation of wine SO_2_ (*O*_2_ *not SO*_2_). **(A)** Acetaldehyde, **(B)** diacetyl, **(C)** isobutyraldehyde, and **(D)** phenylacetaldehyde.

The relationship between the total content in acetaldehyde found in the different samples of each wine and their levels of *O*_2_ *not SO*_2_, is given in Figure 4A. The figure splits wines attending to their age. Levels of acetaldehyde in samples derived from young wines (1-year-old bottled wines) were under 19 mg L^−1^, regardless of the O_2_ delivered to the sample. By contrast, levels in samples derived from aged wines increased with oxidation becoming in all cases above 25 mg L^−1^ in R3 samples. Within each category there are also some differences. In young wines, acetaldehyde levels slightly increased up to 5.8 mg L^−1^ with oxygen exposure only in the wine MF. In aged wines, increases were most noticeable at low O_2_ exposure (R1) and only in the wine BL there was a strong increase at the highest exposure (R3).

Taking into account that once SO_2_ has been depleted, H_2_O_2_ oxidizes mostly ethanol to yield acetaldehyde (Danilewicz, 2013) (Figure 1), it is possible to estimate the amount of acetaldehyde theoretically formed by wine in each sample. The comparison of this expected amount with the real amount of acetaldehyde accumulated in each case will provide an estimation of the fraction of acetaldehyde reacted with polyphenols or further oxidized to acetic acid. As an example, the wine BL consumed 32.4 mg L^−1^ of *O*_2_ *not SO*_2_ in R3. This amount should have produced 32.4/32 = 1.01 mM of H_2_O_2_, which in turn will have oxidized 1.01 mM of ethanol to produce the same molar amount of acetaldehyde: 44.6 mg L^−1^. Since the measured increase of acetaldehyde is 17.7 mg L^−1^ and the increment of acetic acid was null, it can be inferred that 60.4% of the acetaldehyde formed reacted most likely by forming ethyl bridges with polyphenols and that 39.6% of acetaldehyde remained unreacted in the wine.

These calculations are summarized in Table 3. As can be seen, differences between aged and young wines are very high, even taking into account that in some cases the small changes observed in levels of acetaldehyde and the large changes in total SO_2_ cause a large imprecision. The unreacted fraction in old wines was in all cases above 80% in the low oxygen exposure experiment, while in three of the young wines the unreacted fraction was close to zero. A negative percentage means that more acetaldehyde is reacting than it is being formed. A second observation is that the unreacted fraction decreases with the extent of the O_2_ exposure, so that, in the high O_2_ exposure experiment, the unreacted fraction in aged wines was smaller than 25% in three of the wines.

**Table 3:** Fraction of unreacted acetaldehyde (in %) remaining in the wines during the oxidation.

	**Aged wines**				**Young wines**			
**O_2_ exposure**	**SL (%)**	**TS (%)**	**BL (%)**	**CH (%)**	**MF (%)**	**TP (%)**	**HV (%)**	**BS (%)**
Low	135.7 ± 21.6	155.6 ± 44.0	101.4 ± 16.8	87.0 ± 15.0	33.7 ± 8.8	5.9 ± 4.7	4.7 ± 6.1	−2.6 ± 6.8
Medium	60.0 ± 3.2	40.6 ± 2.4	37.4 ± 5.2	45.1 ± 2.3	26.4 ± 2.7	1.6 ± 1.3	−8.3 ± 2.5	−8.6 ± 2.8
High	21.4 ± 1.9	18.0 ± 1.2	39.6 ± 3.3	23.0 ± 0.8	14.8 ± 1.3	0.5 ± 1.0	−2.1 ± 1.5	−1.3 ± 1.6

**Figure 5:**
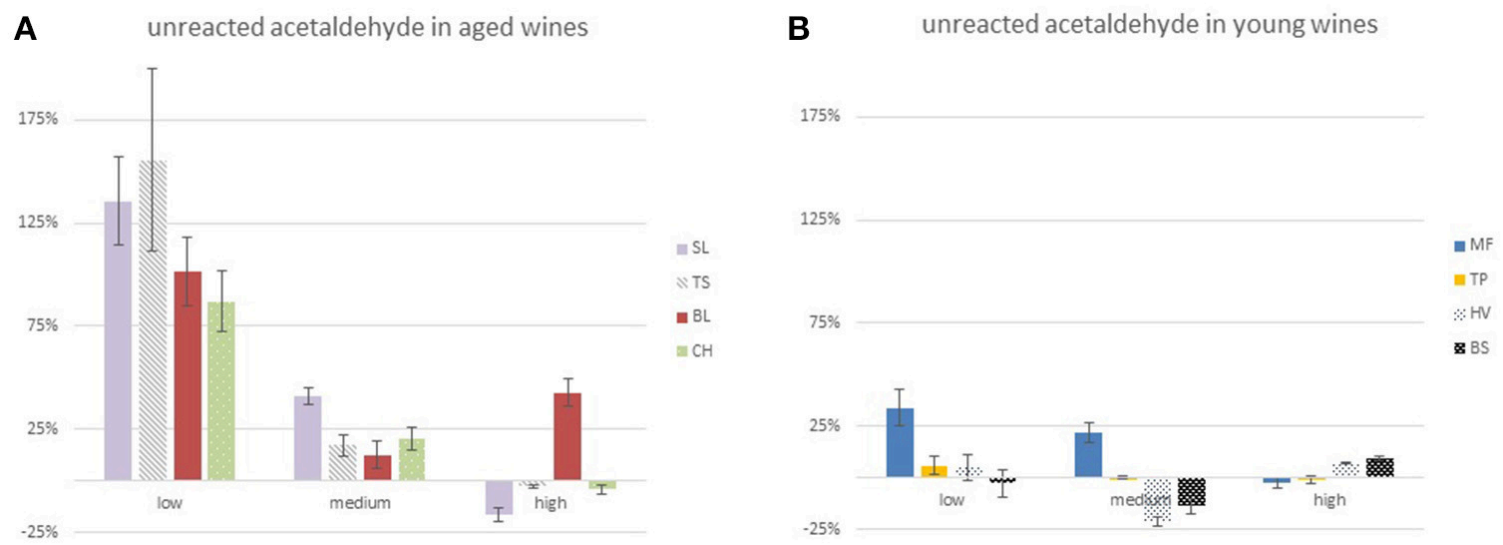
Evolution of the fraction of unreacted acetaldehyde produced by oxidation of ethanol in the three the “virtual”oxidation steps. **(A)** Aged wines and **(B)** young wines.

The effect of the level of O_2_ on the unreacted fraction of acetaldehyde becomes more evident by assuming that the oxidation process can be segmented into three “virtual” steps (first 2.8–10 mg L^−1^; from 15 to 19 mg L^−1^; from 29 to 34 mg L^−1^ of *O*_2_ *not SO*_2_, second column of Table 2), neglecting the fact that the experiments were not consecutive but independent. For instance, the amount of acetaldehyde produced in the second virtual step was estimated by subtracting the levels of acetaldehyde found in R1 from those found in R2, while differences in *O*_2_ *not SO*_2_ between both experiments were used as estimators of the amount of *O*_2_ *not SO*_2_ consumed in such virtual second step. The unreacted fraction of acetaldehyde in those three steps is represented in Figure 5A for aged wines, showing that the unreacted fraction decrease was progressive for SL, TS and CH (quite dramatic in the case of TS) while BL showed a unique increase in the unreacted fraction in the 3rd step. Regarding young wines, shown in Figure 5B, the unreacted fraction of TP was close to 0 in all cases, while for HV and BS became negative in the second period. MF followed a completely different pattern, halfway to those of aged wines.

The previous patterns can be explained attending to three concepts:

1. the ability of acetaldehyde to form strong and stable unreactive complexes with SO_2_
2. the evolution of SO_2_ during oxidation
3. the reactivity of acetaldehyde to the specific profile of aldehyde-reactive polyphenols (ARPs) present in the wines

Attending to those concepts, the parameters which should determine the evolution of acetaldehyde in a specific wine are:

1. the wine content of SO_2_ and of SO_2_ binders
2. the wine content of ARPs
3. the relative rates at which H_2_O_2_ and SO_2_ are formed and consumed, respectively

The higher accumulation of acetaldehyde in aged wines can be explained attending to the much lesser amounts of ARPs which these wines should contain, because of their potentially larger previous exposure to O_2_. Similarly, the higher accumulation of acetaldehyde in the 1st oxidation period observed in Figure 5A can be explained attending to the presence of some remaining SO_2_ in the media. As complexes SO_2_-acetaldehyde are the strongest amongst carbonyls (de Azevedo et al., 2007), newly formed acetaldehyde could displace weak SO_2_-binders from their associations, becoming itself protected from reaction with the limited fraction of ARPs present in aged wines. In the second and third period, however, SO_2_ is progressively depleted, so that newly formed acetaldehyde cannot be further protected from ARPs. The wine-specific patterns seen in Figure 5A should be attributed to their differences in initial SO_2_ and in SO_2_ binders. For instance, the highest fraction of unreacted acetaldehyde of TS in 1st period corresponds to its highest content in total and free SO_2_ (see Table 1). Similarly, the highest fraction observed in the 2nd period for SL is in agreement with its highest content in total SO_2_ and highest level in SO_2_ binders (manifested in its low free SO_2_). The unique behavior of BL wine may be attributed to a possible exhaustion of ARPs. In this context it may be noteworthy that this wine had the lowest TPI and Folin indexes amongst aged wines. In the case of young wines (Figure 5B), the negative fraction of unreacted acetaldehyde of HV in the 2nd period may be related to its lowest level of SO_2_ and may be also to its highest TPI and Folin amongst young wines.

### Clues about the nature of ARPs

A first insight about the nature of ARPs can be obtained by modeling the unreacted fraction of acetaldehyde measured in the different experiments vs. the initial wine chemical composition. Models (summarized in Table 4) were obtained for the different oxidation doses and were in most cases highly explicative, with explained variances by cross-correlation above 94%, except for the high oxygen dose.

**Table 4:** PLS models relating the fraction of unreacted acetaldehyde to the wine initial chemical composition in the different real and virtual oxidation steps.

	**Low**	**Medium**	**Medium (virtual)**	**High**
*R*^2^	0.9642	0.9921	0.9976	0.9235
*R*^2^ cross-validation	0.9417	0.9513	0.9736	0.8729
RMSE	11.1317	2.1824	0.9394	3.7700
RMSE cross-validation	16.2314	6.1868	3.5543	5.5537
PCs	1	3	3	2
B0	65.1787	24.2764	9.7435	14.2498
Combined SO_2_	16.089	11.975	14.472	
Epigallocatechin	14.068			
Epicatechin-3-*O*-gallate		−5.956	−6.974	−7.006
Malvidin-3-*O*-glucoside	−15.809	−5.271	−7.167	−7.269
Malvidin-3-*O*-(6-p-coumaroyl)glucoside				−4.407
Catechin-ethyl-Malvidin	−18.560	−11.535		
Peonidin-3-*O*-glucoside-4-vinylguaiacol	−13.729			
mDP				6.330
% Galloylated tannins		−6.319	−11.857	

In the low exposure case virtual and real are the same.

*RMSE, Root Mean Square Error; PCs, Principal Components used for building the PLS model*.

In general, models give a very large positive coefficient to combined SO_2_ and negative coefficients for nearly all the other components, which suggests that the accumulation of acetaldehyde is the result of the balance between its ability to displace SO_2_ from its adducts with weaker SO_2_-binders and the rate at which it reacts with ARPs.

Attending to the first model, derived from R1 samples, ARPs are mainly anthocyanins: malvidin 3-*O*-glucoside, the vinylguaiacol derivative of peonidin, and the catechin-ethyl-malvidin dimmer. The positive coefficient for epigallocatechin may be related to recent evidences indicating that epigallocatechin is oxidized with a concomitant high SO_2_ consumption (Carrascón et al., 2018) which would cause a shortage of free SO_2_, a concomitant increase of H_2_O_2_ and a peak in acetaldehyde production which could not completely react with ARPs, and instead would displace other SO_2_-binders to form 2-hydroxyethylsulfonate.

In the medium O_2_ exposure level or in the virtual second step, which takes place under small levels of free SO_2_, malvidin 3-*O*-glucoside, galloylated tannins, and epicatechin-3-*O*-gallate have negative coefficients in the model, suggesting that they are the main ARPs for the newly formed acetaldehyde in this stage of oxidation.

Modeling the high exposure level has been more complicated because of the unique behavior of sample BL. Additionally, the virtual step could not be modeled because the predictive ability of the initial chemical composition is limited since wines already have suffered a deep chemical change. Nevertheless, the model is consistent with previous observations, and suggests that under large O_2_ exposure conditions, the weight of SO_2_ is null and that the accumulation of acetaldehyde is basically the result of the balance between its formation and its reaction to form ethyl-bridged structures. In this oxidation step, tannins do not seem to be kinetically critical, and only mDP (medium Degree of Polymerization) has a positive coefficient with the unreacted fraction of acetaldehyde. This would suggest that large tannins are less efficient at forming ethyl bridged structures, and that kinetically, small tannins would be favored.

### Dynamics of wine oxidation when free SO_2_ levels are fading

**Figure 6:**
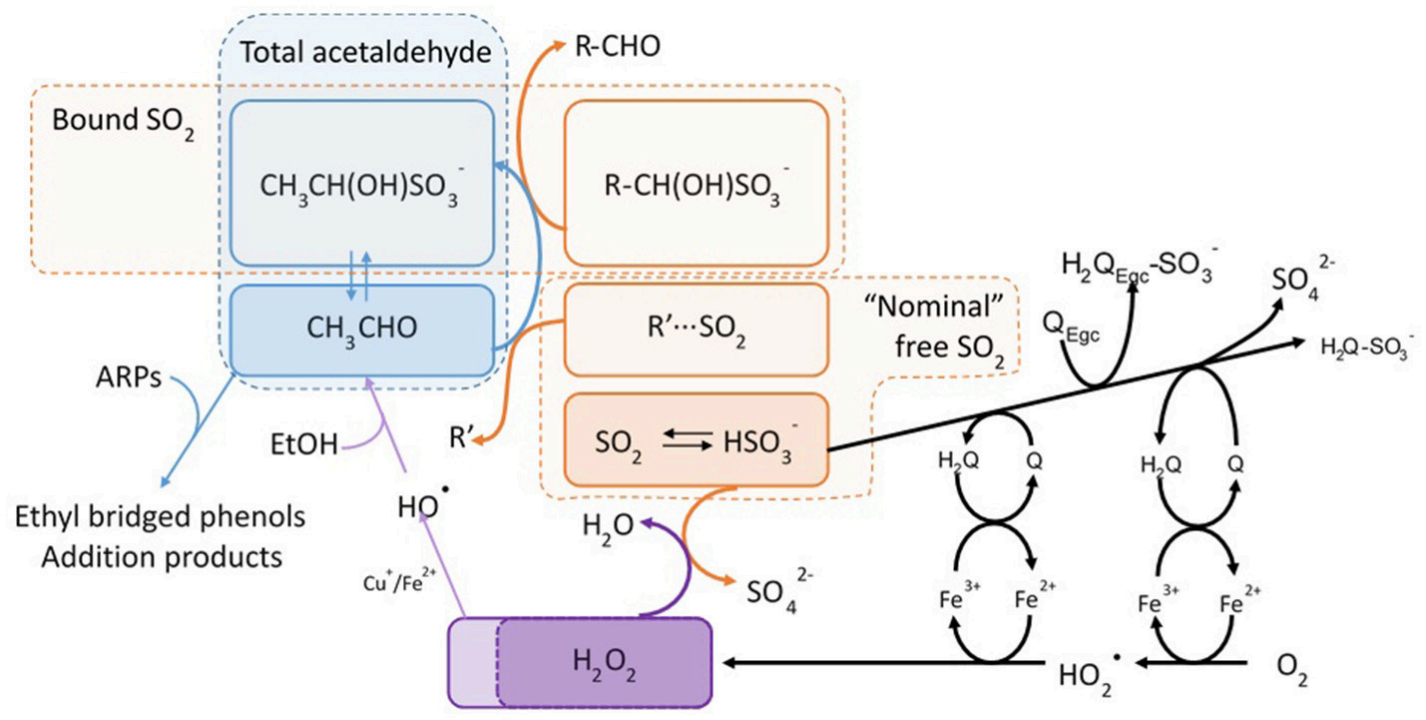
Scheme showing the main elements determining the outcome of wine oxidation when free SO_2_ levels are low.

The previous discussion suggests that when SO_2_ levels become very low, as will inevitably happen in some moment of wine aging, the effects of oxidation will be the result of the relative rates of some key chemical processes, as illustrated in Figure 6.

In the first place, the rate at which ethanol is oxidized to form acetaldehyde depends on the following balance

1. The rate of accumulation of H_2_O_2_, basically related to the rate at which wine takes O_2_
2. The rate at which SO_2_ eliminates H_2_O_2_, critically dependent on the level of truly free SO_2_, which is in turn dependent on:
  - the SO_2_:O_2_ molar ratio associated to the oxidation of polyphenols
  - the amount and nature of SO_2_ complexes

In the second place, the fate of the acetaldehyde formed will depend on:

1. The rates at which acetaldehyde react to wine ARPs, critically dependent on the nature and concentration of those ARPs
2. The rates at which acetaldehyde displaces weaker SO_2_-binders from their combinations

The figure also highlights the relationships between aldehydes, ARPs and SO_2_. The exhaustion of free SO_2_ will have as consequence first the cleavage of the relatively weak associations between anthocyanins and SO_2_, and these anthocyanins will further react with acetaldehyde. Once weak SO_2_ binders have been consumed, the other SO_2_ binders will be released from weakest to strongest. Some of the binders are Strecker aldehydes which once released will change wine aroma (Bueno et al., 2016). Some others, such as diacetyl, are reactive compounds which can then promote different chemical changes.

### The case of diacetyl

The evolution of diacetyl with the progress of oxidation is presented in Figure 4B and again, there is a neat difference between young and aged wines. Levels of diacetyl in aged wines were low, strictly inversely proportional to wine age, and quite stable. This result already suggests that diacetyl slowly but continuously reacts with different wine components during aging in processes more related to time than to the presence of oxygen. Among aged wines, only in TS there is a significant decrease (*p* ≤ 0.05) during oxidation (Table 2). In strong contrast, diacetyl in young wines followed a clear inverse sigmoid characterized by a flat initial period, a strong decrease in the medium O_2_ exposure samples and a last flat step. The decrease was proportional to the initial level and in all cases final diacetyl contents of the wine seemed to stabilize at around 40% of its initial content.

Diacetyl is a quite reactive molecule which can react with a broad range of organic molecules. It has been reported that diacetyl can react with ARPs, although at a much slower rate than acetaldehyde (Blanco-Vega et al., 2011). It can also react to thiols, such as cysteine, producing a wide range of subproducts (Marchand et al., 2000, 2011). Remarkably, one of the reactions suffered by diacetyl and other α-dicarbonyls is Maillard reaction with amino acids, one of whose possible outcomes is the Strecker degradation to form the corresponding Strecker aldehydes (Oliveira et al., 2011a). The possible implication of diacetyl in the formation of these components will be discussed later. The flat initial period observed in Figure 4B can be explained because in the low oxygen exposure conditions, diacetyl would be mostly complexed with SO_2_ (Bueno et al., 2016), and would be protected. At higher O_2_ exposure levels, however, unprotected diacetyl would react with available nucleophiles. The last flat period may be attributed to the exhaustion of such nucleophiles.

### Strecker aldehydes

**Figure 7:**
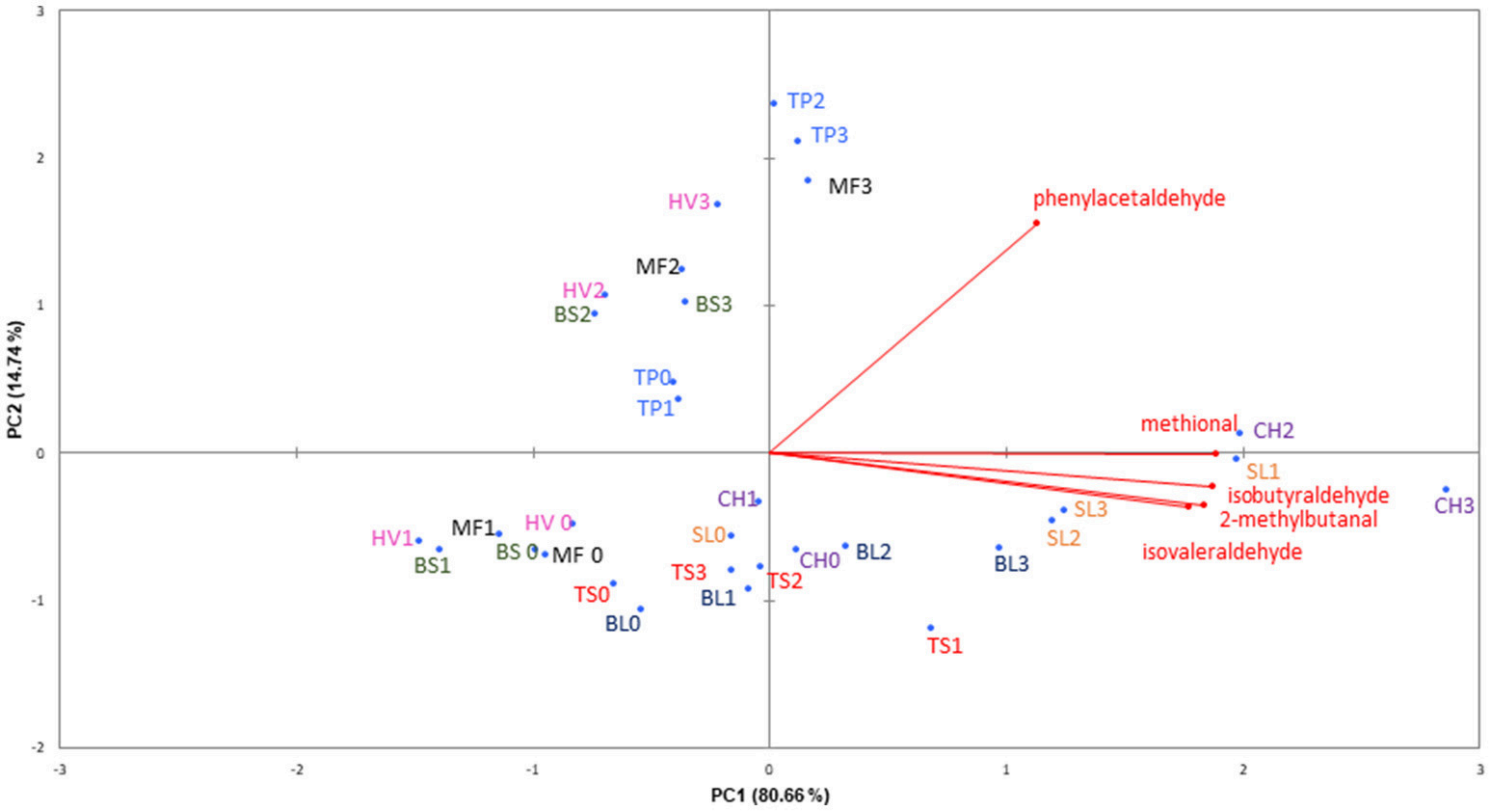
PCA plot with data from Strecker aldehydes normalized by the consumed oxygen not invested in the oxidation of sulfur dioxide.

The five Strecker aldehydes accumulate during wine oxidation in all wines, as can be seen in Table 2. As previously observed, the accumulation takes place at relatively high consumptions of oxygen (Bueno et al., 2016), and in most wines it was observed only when the wine was exposed at least to medium amounts of oxygen (R2). The relationship between levels of Strecker aldehydes in the different wines and levels of oxygen “not invested in the oxidation of sulfur dioxide” can be seen in the Principal Component plot shown in Figure 7. The plot, which retains more than 95% of the original variance, shows that non-aromatic aldehydes (methional, isobutyraldehyde, 2-methylbutanal, and isovaleraldehyde) follow a quite similar and correlated behavior, while phenylacetaldehyde follows a quite distinct pattern.

Regarding samples, the PCA plot reveals that samples are largely segregated by its age: older wines are placed in the central and right south parts of the plot, while young wines are in the left and upper part of the plot. Older wines displace strongly to the right along the first component and just slightly up along the second when they oxidize, indicating that these compounds form comparatively more methional, isobutyraldehyde, 2-methylbutanal, and isovaleraldehyde than phenylacetaldehyde. The plot also shows that the magnitude of the change suffered by aged wines is related to the position of the original wine in the plot: the further right the original samples are, the further right are displaced the samples exposed to higher levels of oxygen. This implies that in these older wines the amount of aldehyde formed is proportional to the amount of aldehyde already present in the wine. This makes sense, since the history of previous exposure to oxygen of older wines is large, indicating that their initial content maybe strongly influenced by their specific ability to form aldehydes.

Quite differently, younger wines when oxidize displace north and slightly right, indicating that these wines tend to form higher levels of phenylacetaldehyde than of the other Strecker aldehydes. Moreover, the plot basically separates initial and low O_2_ exposure samples (R0 and R1) from those of medium and high exposure (R2 and R3), indicating that phenylacetaldehyde formation takes part preferably at medium O_2_ exposure. Additionally, the magnitude of the change in these cases does not seem to be related to the initial position of the wine in the plot, meaning that the aldehyde content of the initial wine seems to be a poor predictor of its ability to produce aldehydes upon oxidation.

The more specific differences in oxidation patterns between samples can be appreciated with the plots in Figure 4. Isobutyraldehyde, in Figure 4C, exemplifies the patterns followed by methional, 2-methylbutanal, and isovaleraldehyde, and shows that levels of aldehyde in older samples steadily increases with oxygen exposure and that increases are proportional to the original content of the wine. By contrast, levels in young wines increase just slightly and only become significant at high O_2_ exposure levels. It is worth mentioning that samples in the plot are nearly perfectly arranged by age. This, as was previously mentioned for diacetyl, could be a clue indicating that time, and not only oxygen, plays a role in the accumulation of these components. Phenylacetaldehyde, in Figure 4D, follows a quite different pattern. The four young wines follow a clear sigmoidal pattern of accumulation, indicating that the formation of phenylacetaldehyde took specifically place when the wine was exposed to medium levels of oxygen. Higher or lower levels did not have any additional effect on the levels of this aldehyde. The resemblance with the plot seen for diacetyl in Figure 4B is remarkable. Older wines, by contrast, follow a pattern quite similar to the one observed for isobutyraldehyde in Figure 4C; levels increase steadily with level of oxygen, although, less evidently for SL, the formation was yet more intense when samples were exposed to medium levels of oxygen.

The segregation between aged and young wines shown in the PCA plot in Figure 7 is consistent with the assumption that Strecker aldehydes, except phenylacetaldehyde, are also subject to reaction with ARPs. Aged wines, which were found to contain less ARPs are the ones with higher increases in aldehydes. The odd pattern followed by phenylacetaldehyde, which strongly accumulates in young wines, could be simply related to a reduced reactivity toward ARPs because of steric reasons. The reactivity of the different Strecker aldehydes toward ARPs is not known and should be the subject of further research.

### Clues about the origin and fate of strecker aldehydes

The accumulation of Strecker aldehydes in older wines is strongly and significantly correlated to the levels of precursor amino acids in wine: (R = 0.98; 0.96; 0.99; 0.99, and 0.96 for the pairs valine-isobutyraldehyde, leucine-isovaleraldehyde, isoleucine-2-methylbutanal, methionine-methional, and phenylalanine-phenylacetaldehyde). In younger wines, however, only the correlations between the pairs isoleucine-2-methylbutanal and phenylalanine-phenylacetaldehyde, are significant (R = 0.99 in both cases). The lack of significance in the other cases may be attributed to the low variability of their corresponding amino acids (<4 mg L^−1^, with RSD inferior to 16% in all cases) and to the low levels of aldehyde accumulated.

The amount of aldehyde accumulated by unit of amino acid originally present in wine is in all cases strongly linked to wine age, being the effect of age in phenylacetaldehyde opposite to that observed in the other aldehydes. Young wines accumulated 9.2 μg L^−1^ of phenylacetaldehyde per mg L^−1^ of phenylalanine present, while just 3.0 μg L^−1^ per mg L^−1^ were observed in aged wines. Conversely, for 2-methylbutanal, aged wines accumulated 1.3 μg L^−1^ of aldehyde, while young wines just 0.17 μg L^−1^ per mg L^−1^ of amino acid. If such difference was exclusively due to the effect of ARPs, that would mean that in young wines, more than 85% of aldehyde formed has been removed by reaction.

Levels of diacetyl are negatively and significantly (*P* < 0.05 in all cases) correlated to the accumulation of non-aromatic Strecker aldehydes. This may suggest that diacetyl reacts with the amino acid precursor to form different species. i.e., although diacetyl can induce the Strecker degradation of amino acids (Rizzi, 2006), it can also form a wide range of heterocyclic products belonging to chemical classes such as oxazoles, pyrazines, pyrroles, or pyridines (Piloty and Baltes, 1979; Pripis-Nicolau et al., 2000).

Further clues about the nature and relative importance of the chemicals involved in the accumulation of Strecker aldehydes can be extracted from the PLS models summarized in Table 5. The eight models for non-aromatic aldehydes closely follow the same structure, and in all cases attribute a positive weight to the amino acid, higher at high levels of oxygen (Table 5A), and negative roles to anthocyanins, which would act as ARPs. Some other elements in the models are worth mentioning. First, in the case of isovaleraldehyde in Table 5A, the negative role of diacetyl, which would compete for the amino acid, as previously suggested. Second, iron has positive coefficients in some cases, suggesting that it may play a role as catalyst. Third, in the medium oxygen exposure (Table 5B), the negative coefficients for procyanidins and catechin in tannins suggest that they act also as ARPs.

**Table 5:** PLS models relating the increase in total Strecker aldehyde, normalized by the consumed O_2_ not invested in the oxidation of SO_2_ in each wine, to the initial composition of the wines: **(A)** R3-R0 and **(B)** R2-R0.

**A**					
**R3-R0**	**Isobutyraldehyde**	**Isovaleraldehyde**	**2-Methylbutanal**	**Methional**	**Phenylacetaldehyde**
*R*^2^	0.968	0.995	0.9997	0.990	0.916
*R*^2^ cross-validation	0.924	0.982	0.9994	0.964	0.821
RMSE	1.558	1.592	0.108	1.863	8.793
RMSE cross-validation	2.737	3.402	0.178	3.946	14.685
PCs	2	4	3	3	2
B0	16.564	16.193	8.683	23.599	67.033
Diacetyl		−6.680			
Valine	5.304				
Leucine		15.509			
Isoleucine			4.314		
Methionine				7.684	
Phenylalanine					12.307
Prodelphinidins					6.587
Catechin in tannins					8.574
Malvidin-3-*O*-glucoside	−4.088	−5.178		−8.521	
Petunidin-3- *O*-glucoside			−2.208	−7.336	
Delfinidin-3- *O*-glucoside			−1.951		
Peonidin-3-*O*-glucoside-4-vinylguaiacol	−2.029		−1.700	−5.849	
Petunidin-3-*O*-glucoside-4-vinylguaiacol		−4.621			
Fe	2.506			5.489	18.349
**B**					
**R2-R0**	**Isobutyraldehyde**	**Isovaleraldehyde**	**2-Methylbutanal**	**Methional**	**Phenylacetaldehyde**
*R*^2^	0.849	0.985	0.929	0.957	0.957
*R*^2^ cross-validation	0.717	0.966	0.868	0.863	0.780
RMSE	2.481	2.288	1.285	2.902	4.414
RMSE cross-validation	3.885	3.929	2.003	5.917	11.391
PCs	2	3	2	2	3
B0	8.564	11.421	5.595	16.308	55.455
Diacetyl					13.972
Valine	0.389				
Leucine		7.878			
Isoleucine			1.729		
Methionine				2.604	
Phenylalanine					14.205
Procyanidins	−1.731			−5.817	
Catechin in tannins	−1.950				1.624
Malvidin-3-*O*-glucoside	−1.761	−6.822	−1.560	−4.668	
Petunidin-3- *O*-glucoside				−3.388	
Malvidin-3- *O*-(6-p-coumaroyl)glucoside		−5.372	−1.210		
Peonidin-3-*O*-glucoside-4-vinylguaiacol	−2.870	−8.405	−1.676		
Petunidin-3-*O*-glucoside-4-vinylguaiacol			−1.188	−3.405	
Fe				2.455	9.938

*RMSE, Root Mean Square Error; PCs, Principal Components used for building the PLS model*.

Models for phenylacetaldehyde are completely different. First, because anthocyanins do not have any weight, and second, because it is the single aldehyde for which tannins have positive weight. This may suggest that quinones derived from prodelphinidins and catechin in tannins would be particularly reactive toward phenylalanine yielding phenylacetaldehyde. This is consistent with the observation that *o*-quinones at pH 7 with K_3_Fe(CN)_6_ favor Strecker production of phenylacetaldehyde over methional (Rizzi, 2006). The higher levels of these quinones present in young wines, together with the negligible reactivity toward ARPs, would explain the higher formation of this aldehyde in young wines. A third specificity of phenylacetaldehyde is the large and positive coefficient taken by diacetyl in Table 5B, suggesting that in this particular case, diacetyl is one of the α-dicarbonyls inducing the Strecker degradation of phenylalanine. The distinct pattern followed by phenylacetaldehyde has been already observed (Rizzi, 2006; Grant-Preece et al., 2013).

### Fusel alcohols as precursors of strecker aldehydes

Models in Table 5 suggest that fusel alcohols are less relevant than amino acids as precursors for Strecker aldehydes, in agreement with previous studies (Grant-Preece et al., 2013; Ferreira et al., 2014; Bueno et al., 2016). The potential relevance of fusel alcohols can be further assessed by estimating the proportion of alcohol oxidized, assuming that they will be oxidized at extents similar to those of ethyl alcohol. The molar fractions of ethanol oxidized were estimated from the expected levels of H_2_O_2_ produced and are given in Table 6. As can be seen, ethanol oxidizes by percentages which range between 0.003 and 0.045%, depending on the alcoholic degree of the wine and on the oxygen exposure. If equivalent amounts of isobutanol, 2-methylbutanol, isoamyl alcohol, methionol, and β-phenylethanol have been oxidized to form the corresponding aldehydes, data show that only in the cases of isobutyraldehyde, 2-methylbutanal and isovaleraldehyde, the direct oxidation of the precursor alcohol can be a significant source of those aldehydes. Taking into account, however, the large fraction of aldehyde removed by reaction with ARPs and the fact that levels of alcohols tend to be less variable than those of amino acids, the contribution of the alcohols to the specific ability of a sample to form these aldehydes should be quite limited. In the case of methional, the oxidation of methionol may account just for a marginal amount of the aldehyde accumulated, in accordance with previous observations (Bueno et al., 2016). In the case of phenylacetaldehyde and taking into account its low reactivity toward ARPs, the oxidation of the alcohol as source of aldehyde should not be discarded, particularly in wines containing high amounts of this alcohol.

**Table 6:** Fraction of Strecker aldehyde formed in wine which could be attributed to the oxidation of the corresponding precursor alcohol, assuming that this one oxidizes in the same proportion than ethanol in the Fenton reaction.

	**H_2_O_2_ produced (mMol)**	**Ethanol (Mol)**	**Fraction of oxidized ethanol (%)**	**Isobutanol (mg L^−1^)**	**Formed isobutyraldehyde explained (%)**	Isoamyl alcohol^*^ (mg L^−1^)	**Isovaleraldehyde + 2-methylbutanal explained (%)**	**Methionol (mg L^−1^)**	**Methional explained (%)**	**β-Phenylethanol (mg L^−1^)**	**Phenylacetaldehyde explained (%)**
SL R1	0.09	2.31	0.0040	36.49	>100	248.04	>100	2.15	1	38.74	18
SL R2	0.46		0.0200		69		>100		2		36
SL R3	0.93		0.0402		73		>100		3		50
TS R1	0.09	2.48	0.0035	23.15	>100	183.89	>100	0.97	>100	20.60	>100
TS R2	0.51		0.0204		70		>100		2		29
TS R3	1.06		0.0425		79		>100		3		47
BL R1	0.17	2.31	0.0072	34.05	69	236.81	>100	1.91	2	35.77	21
BL R2	0.59		0.0256		77		>100		2		22
BL R3	1.01		0.0438		67		>100		2		29
CH R1	0.22	2.40	0.0092	37.11	>100	253.14	>100	2.31	2	34.13	13
CH R2	0.59		0.0247		41		87		1		10
CH R3	1.07		0.0446		39		>100		1		16
MF R1	0.20	2.31	0.0088	45.58	>100	224.51	>100	2.15	>100	44.45	72
MF R2	0.54		0.0232		>100		>100		6		13
MF R3	0.89		0.0387		>100		>100		8		16
TP R1	0.15	2.57	0.0058	23.10	>100	185.28	>100	0.99	>100	20.70	>100
TP R2	0.54		0.0209		>100		>100		2		6
TP R3	0.96		0.0374		>100		>100		3		12
HV R1	0.30	2.48	0.0122	32.82	>100	216.65	>100	1.86	>100	33.52	>100
HV R2	0.60		0.0243		>100		>100		19		12
HV R3	1.01		0.0405		>100		>100		10		14
BS R1	0.27	2.31	0.0118	36.40	>100	243.19	>100	1.91	>100	34.36	>100
BS R2	0.59		0.0254		>100		>100		12		13
BS R3	1.00		0.0433		>100		>100		8		20

* *Sum of 2-methylbutanol and isoamyl alcohol*.

### Patterns of accumulation of aldehydes and potential sensory effects

Attending to the previous discussion, the patterns of accumulation of Strecker aldehydes (Figures 4C,D) can be explained attending to the availability of reactive quinones able to induce the Strecker degradation of the amino acid precursors and to the presence of ARPs. In the first stage of the oxidation, quinones surely will be poorly available because they will be reacting fast with SO_2_ or with other competing nucleophiles, so that Strecker degradation of amino acids will not be intense. In addition, the few molecules of aldehyde formed will react with ARPs, so that non-aromatic Strecker aldehydes do not accumulate, as seen in Table 2 and Figure 4C. In contrast, in the case of phenylacetaldehyde (Figure 4D), which is the least reactive to ARPs, clear increments of the aldehyde in older wines can be observed even in the low O_2_ exposure conditions. The reasons why those increments are not observed in young wines may be related to the highest levels of ARPs of these wines, which would mean that reactivity of phenylacetaldehyde toward ARPs contained in young wines is not null. At medium O_2_ exposure levels, in which SO_2_ becomes poorly available, both plots suggest that there is an intense degradation of amino acids through the Strecker pathway. In the case of non-aromatic aldehydes increases are limited because of the presence of ARPs and because in these cases diacetyl, which is now released from its SO_2_ adducts, would be competing for the amino acids. On the contrary, in the case of phenylacetaldehyde, the huge increases observed in young wines in this specific range of oxygen, should be attributed to the lower fraction consumed by ARPs, to the potential extra contribution of diacetyl as reactive α-dicarbonyl and to the possible specific Strecker degradation of phenylalanine by quinones derived from catechin in tannins. Finally, the flat part of the plot in Figure 4D may be due to the exhaustion of the amino acid precursors.

**Figure 8:**
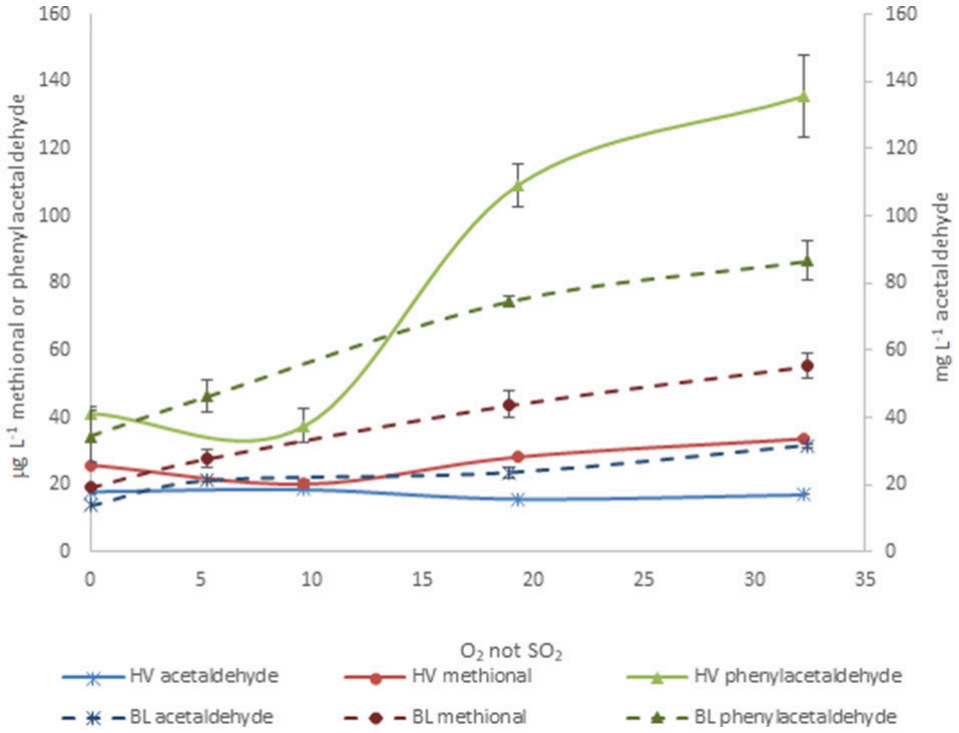
Comparison of the accumulation of acetaldehyde (x), methional (circles), and phenylacetaldehyde (triangles) in aged (dotted lines) and young (filled lines) wines during oxidation.

**Figure 9:**
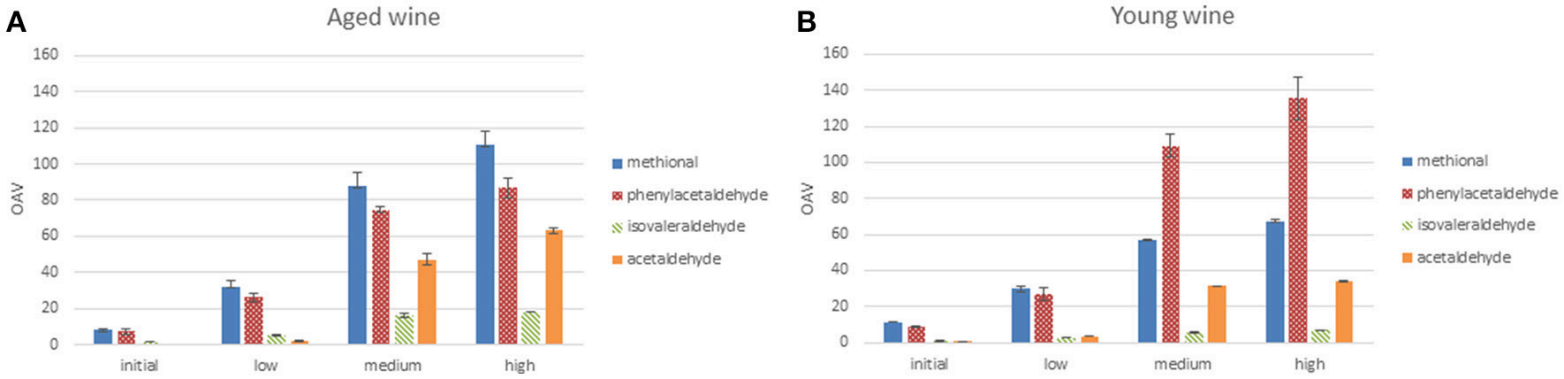
Evolution of the estimated Odor Activity Values (OAVs) of free acetaldehyde and free Strecker aldehydes for the wines **(A)** BL (example of aged wine) and **(B)** HV (example of young wine) during oxidation. Odor thresholds taken from Guth (1997), Escudero et al. (2000b) and Cullere et al. (2007). In initial and low oxygen exposure samples, the proportion of aldehyde under free form has been estimated from the corresponding SO_2_ binding constants (de Azevedo et al., 2007; Bueno et al., 2014, 2016); in R2 and R3, as there was no free SO_2_, all the aldehyde was considered free.

A quite important corollary of all the previous observations and hypotheses, is that regarding the temporal pattern of accumulation of aldehydes, Strecker aldehydes accumulate long before acetaldehyde does. This can be seen in Figure 8, which compares the evolution during oxidation of acetaldehyde, methional and phenylacetaldehyde in a young (HV) and an aged wine (BL). The highest difference between acetaldehyde and Strecker aldehydes is found in the second oxidation stage and is particularly evident in the young wine (filled lines). It can be clearly appreciated that while acetaldehyde decreases, the levels of Strecker aldehydes peak up. In aged wines, even if in the low and high O_2_ exposure levels the three aldehydes increase in parallel, increases of Strecker aldehydes in the medium exposure levels are much higher than those of acetaldehyde. What this implies in sensory terms can be best assessed by estimation of the corresponding Odor Activity Values, as seen in Figure 9. It can be seen than in methional and phenylacetaldehyde reach much higher OAVs than acetaldehyde, and more important, even at low oxygen exposure they reach OAVs above 20, when acetaldehyde is barely detectable.

## Conclusions

The pattern of accumulation of acetaldehyde in red wine during oxidation is related to the wine content in SO_2_ and SO_2_ binders, to the relative rates at which H_2_O_2_ and SO_2_ are formed and consumed, respectively, and to the wine content in ARPs, which attending to our models, should be mostly anthocyanins and small tannins. This explains why in young wines there is hardly any accumulation of acetaldehyde, regardless of the O_2_ consumed by the wine, while in aged wines, acetaldehyde accumulates as long as there remains a little fraction of SO_2_ able to protect it from the reaction with ARPs. Acetaldehyde will further accumulate only when these ARPs have been exhausted.

The accumulation of Strecker aldehydes follows a completely different pattern consistent with a major formation via Strecker degradation of amino acids. Nevertheless, non-aromatic Strecker aldehydes share with acetaldehyde a high affinity toward ARPs, which exert a relevant negative influence on their accumulation and explain the higher levels accumulated in aged wines. The Strecker degradation of non-aromatic amino acids would take place when SO_2_ is poorly available through reactive quinones in reactions likely catalyzed by iron. Diacetyl would be a competitor for those amino acids as its presence is related to reduced formation of the corresponding aldehydes. Phenylacetaldehyde follows a quite distinct pattern likely derived from a much reduced reactivity toward ARPs, to the possibility that diacetyl in this case also induces Strecker degradation, and to the potential higher specificity for the quinones of catechin in tannins.

A final corollary of the differential patterns of accumulation is that Strecker aldehydes will accumulate before acetaldehyde, and in wines containing normal levels of Strecker amino acids will be potentially responsible for sensory changes long before acetaldehyde becomes evident.

## Author contributions

MB, AE, and VF have participated in the experimental design. MB has analyzed the Strecker aldehydes and diacetyl, AM-C has carried out the wine oxidation procedure, the analysis of acetaldehyde and the oxygen measurements under the supervision of AE and VC has done the phenolics determinations following the guidelines of PF-Z. MB has carried out the aldehydes data analysis and its interpretation, VC has built the statistical models and with PF-Z have discussed polyphenol results. AE and AM-C have studied oxygen kinetics and VF has carried out a global evaluation of the oxidation process. MB, VC, and VF have drafted the article. All the authors have collaborated in the critical revision and final approval of the manuscript.

### Conflict of interest statement

The authors declare that the research was conducted in the absence of any commercial or financial relationships that could be construed as a potential conflict of interest. The handling Editor declared a past co-authorship with one of the authors, VF.
