# Supplementary figures and images for: Formation and Accumulation of Acetaldehyde and Strecker Aldehydes during Red Wine Oxidation
Source: Front Chem. 2018 Feb 14;6:20. doi: 10.3389/fchem.2018.00020 (PMC5817066; doi:10.3389/fchem.2018.00020)

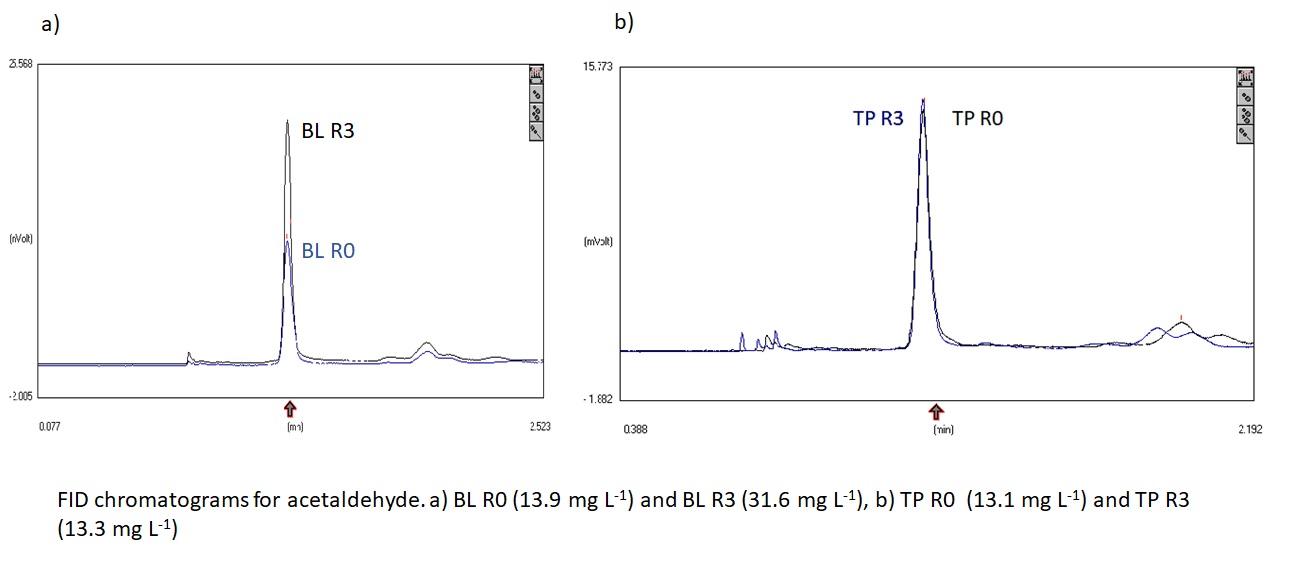

Supplement: Supplementary file 1 [file Image1.JPEG]

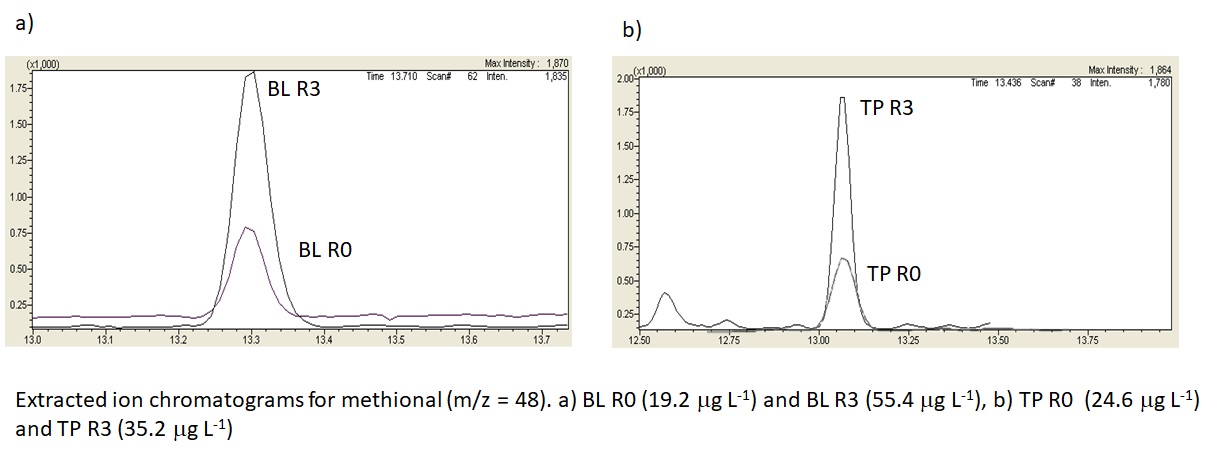

Supplement: Supplementary file 2 [file Image2.JPEG]
